# Groundwater Transport in a Glaciomarine Aquitard: Paleosalinity and Landslide Implications

**DOI:** 10.1111/gwat.70045

**Published:** 2026-01-13

**Authors:** M. J. Hinton, S. Alpay, H. L. Crow

**Affiliations:** ^1^ Natural Resources Canada Geological Survey of Canada Ottawa Ontario Canada

## Abstract

Leaching of marine salinity in the porewater of glaciomarine muds is one precursor to landslide hazard. In this study, groundwater modeling is used to quantify vertical groundwater flow, constrain paleosalinity, and characterize past and future progression of leaching with depth in Champlain Sea sediments. The Breckenridge Creek site, ~15 km northwest of Ottawa, Canada, was cored within a thick sequence (up to 98 m) of Champlain Sea muds that form a regional aquitard in the St. Lawrence Lowlands and Ottawa Valley. Porewater chloride concentrations ([Cl]), up to 12,250 mg/kg, and δ^18^O as high as −7.18‰, indicate remnant seawater. One‐dimensional groundwater transport modeling simulates porewater [Cl] and δ^18^O with depth simultaneously and constrains specific discharge, q, from 2.40 to 2.51 mm/a. Groundwater transport modeling and three‐component mixing of seawater, glacial meltwater and meteoric water constrain the range of initial [Cl] between 14,000 and 15,700 mg/kg (72–80% seawater) and initial δ^18^O between −5.99 and −5.61‰. The glacial meltwater component of Champlain Sea bottom waters at the Breckenridge site has a maximum δ^18^O value of −22.4‰. Downward leaching to the salinity threshold of <2 g/L for geotechnical sensitivity development reached a depth of 20.6 m. Modeling indicates the leaching front currently progresses at a rate of 2.5 m/1000 years, slower than advection of freshwater infiltration because of upward diffusion and dispersion of marine solutes. Notably for landslide hazard, the highest measurements of geotechnical sensitivity coincide with the leached zone.

## Graphical abstract

Transient 1D modeling of both [Cl] and δ^18^O demonstrates that advection and diffusion contribute to solute transport in porewater of glaciomarine Champlain Sea muds. Samples with the highest sensitivity (>30) for landslide hazard occur in the current leached zone.

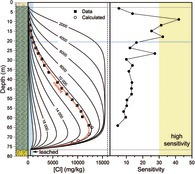

## Introduction

**Figure 1:**
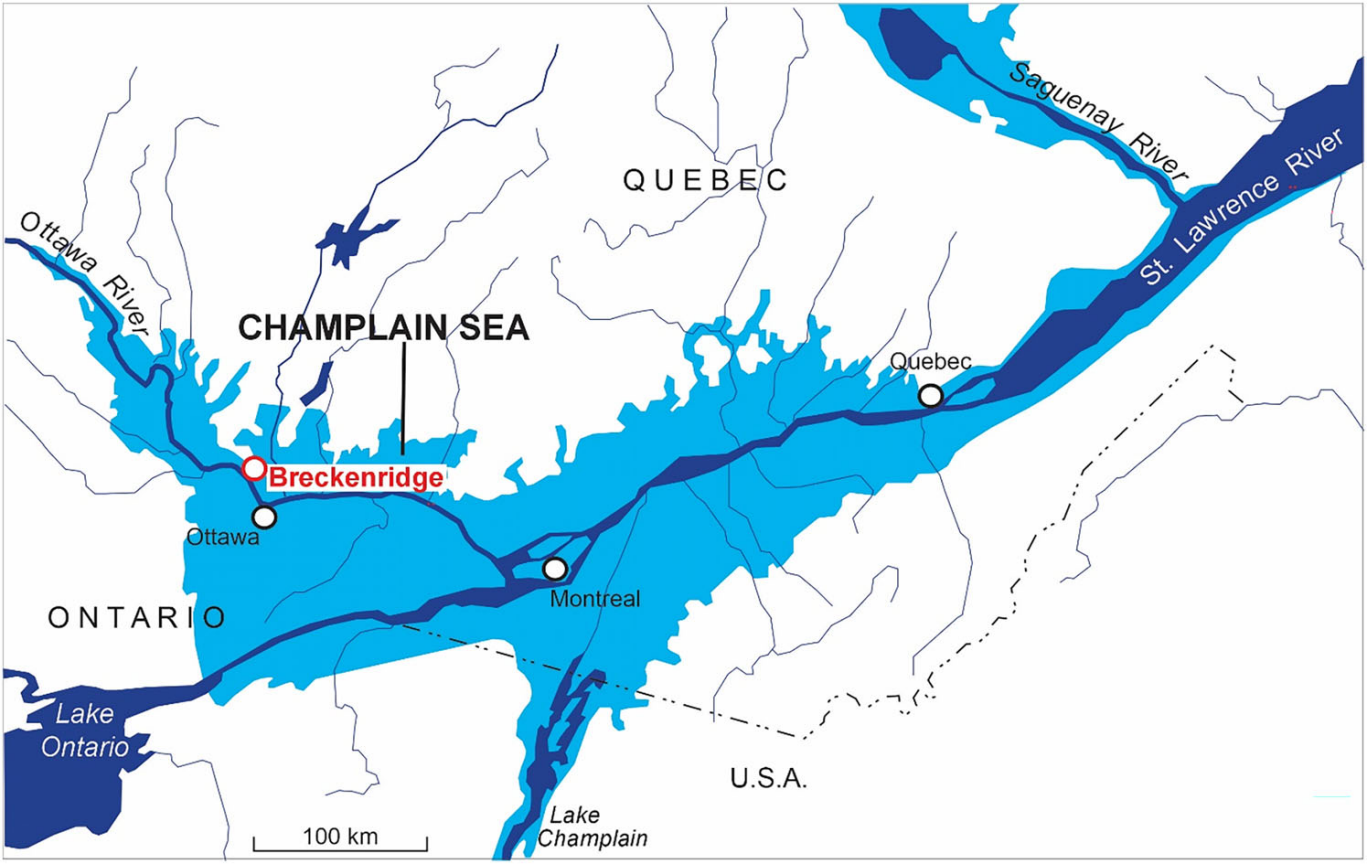
The Breckenridge study site within the diachronous maximal margins of the Champlain Sea in the St. Lawrence Lowlands (after Aylsworth 2012).

Glaciomarine aquitards are prominent in regions where melting and retreat of ice from the last continental glaciation opened a pathway for seawater to inundate isostatically depressed lands (Parent and Occhietti 1988). During retreat of the Laurentide ice sheet at the end of the last glaciation, the Champlain Sea inundated the Ottawa Valley from ~12,800 to 10,400 calendar years before present (BP; Parent and Occhietti 1988; Richard and Occhietti 2005; Lewis and Todd 2019; Figure 1). Mud deposits (silt and clay particle sizes) from the Champlain Sea, known locally as Leda clay (Crawford 1968), form a low permeability, regionally extensive aquitard. The properties of Champlain Sea muds influence ground settlement, landslide and earthquake hazards (amplification of ground motion) and building code provisions (Crawford 1968; Penner and Burn 1978; Carson 1981; Crow et al. 2011; Quinn et al. 2011; Banab et al. 2012; Crow et al. 2014).

Proglacial deposition of glacial muds in saline marine water led to flocculation of silt‐ and clay‐sized particles, which gives the sediment its strength, an open structure, and high water content (Torrance 2017). Subsequent isostatic uplift above sea level allowed infiltration and recharge of freshwater, which gradually displaced saline porewaters from the muds by advection, dispersion, and diffusion. The process of leaching can weaken the flocculated structure, which can transform the sediment to behave as a liquid when disturbed.

Sensitivity is the ratio of undisturbed to remolded compressive strength of sediments. There are various classifications for sensitivity; the Norwegian Geotechnical Society classifies values below 8 as low, between 8 and 30 as medium‐high, and values above 30 as high sensitivity (Norsk Geoteknisk Forening 1974; cited by Torrance 1983). High sensitivity muds with low remolded shear strength are prone to retrogressive landslides in which failure at the toe of the slope removes upslope support, causing successive slope segments to also become unstable and fail. Liquefaction of disturbed high sensitivity sediments can further induce retrogressive landslides to expand rapidly (>1 ha to ~30 km^2^), which can endanger human life and property (Blais‐Stevens 2020).

Several studies report that high sensitivity in sediments develops when porewater salinity falls below a threshold of ~2 g/L (Torrance 1975; Lefebvre and Grondin 1978; Crow et al. 2014). Leaching is considered a precursor to sensitivity development; however, the criterion is empirical. Glaciomarine muds are less susceptible to landslides at the time of deposition; however, geotechnical sensitivity develops over time as porewater becomes less saline with the infiltration of freshwater, in conjunction with changes in geomorphology from isostatic rebound and erosion. Other geochemical and geotechnical factors influence sensitivity development (Torrance 1983); therefore, not all leached sediment necessarily becomes sensitive, whereas some sediments can develop sensitivity at a higher porewater salinity (Duhaime et al. 2013).

Quigley et al. (1983) and Desaulniers and Cherry (1989) determined that diffusion is the major control on ion transport at their study sites in Champlain Sea sediments despite non‐zero measurements of vertical hydraulic gradients. Further, Desaulniers and Cherry (1989) and Cloutier et al. (2010) interpreted the salinity of the Champlain Sea to be approximately one‐third that of seawater (12 g/kg), although micropaleontological and isotopic studies suggest salinities of >30 g/kg (Rodrigues 1988). In this study, analysis and interpretations of porewater chemical profiles in thick Champlain Sea sediments provide enhanced understanding of paleosalinity, solute transport and implications for sensitivity development.

This article presents 1D groundwater transport models of porewater tracers, chloride concentration and δ^18^O, and their evolution in the Champlain Sea sequence with objectives to: (i) quantify solute transport, particularly vertical groundwater flow (specific discharge), (ii) constrain paleosalinity and identify source waters to the Champlain Sea, and (iii) characterize the past and future progression of leaching with depth that provide insight into the effects on sensitivity and potential landslide hazard.

## Study Site

**Figure 2:**
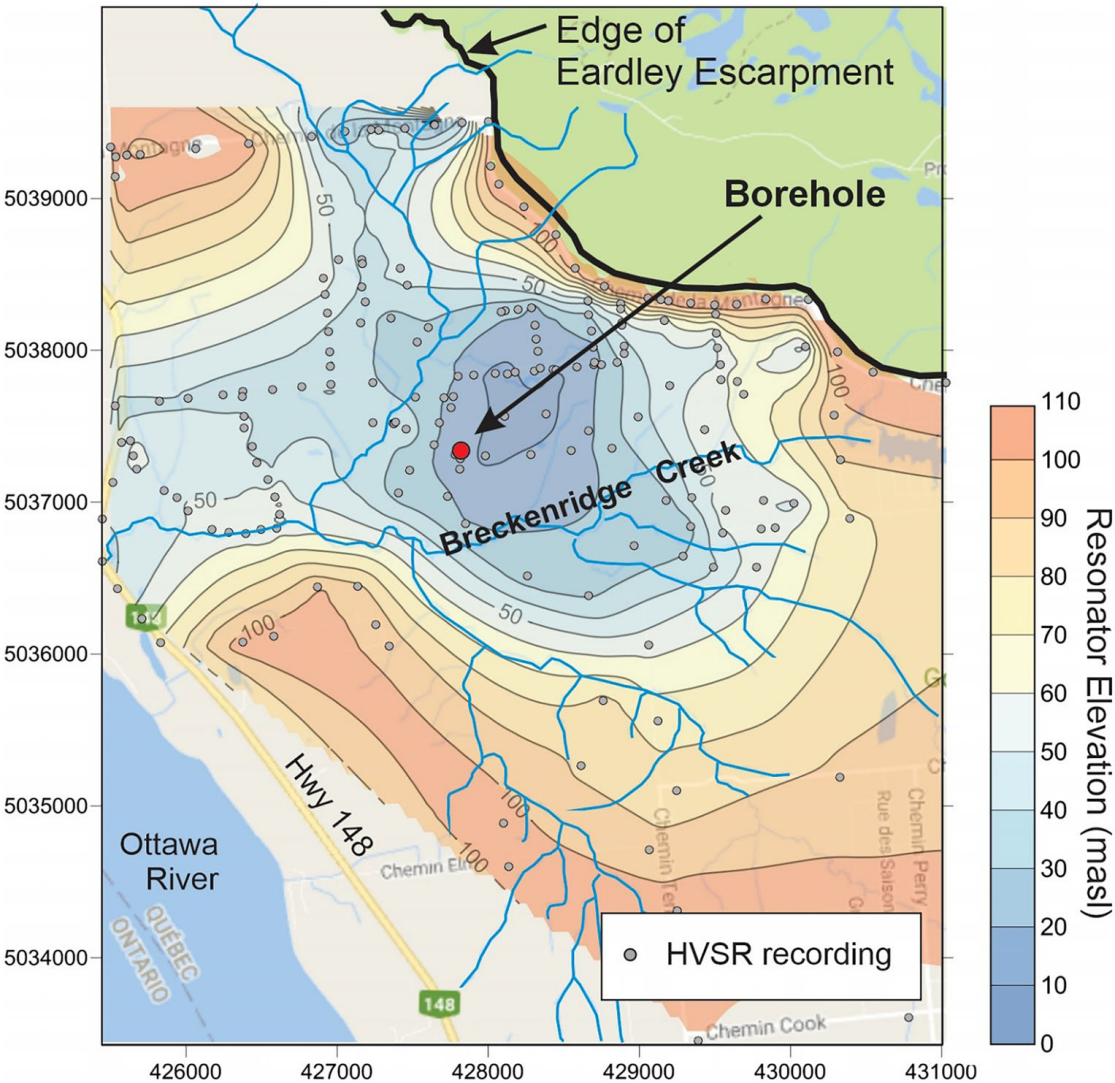
Contour map showing the resonator elevation (till or bedrock) in meters above sea level. Soft sediment thickness reaches 98 m in the deep oval‐shaped basin. The Eardley Escarpment northeast of the borehole is a bedrock outcrop. Gray circles are geophysical measurement sites (after Crow et al. 2017).

The Breckenridge Creek watershed, an agricultural area interspersed with forested valleys, is located ~15 km northwest of Ottawa, Canada, in the Municipality of Pontiac, Quebec. Twenty‐six landslide scars are clustered along the creek and its tributaries in an area of ~11 km^2^ (Figure 1, Brooks et al. 2021; see also Figure S1). Extensive geophysical field work, follow‐up drilling and core sampling identified soft sediments locally in the basin, up to 98 m thick (Figure 2; Crow et al. 2017, 2021), whereas most occurrences of Champlain Sea sediments in the St. Lawrence Lowlands are <20 m in thickness (Parent et al. 2021). Therefore, the Breckenridge field site and borehole provided an optimal location to study a thick Champlain Sea sequence in a regionally extensive glaciomarine aquitard with a history of retrogressive landslides.

**Figure 3:**
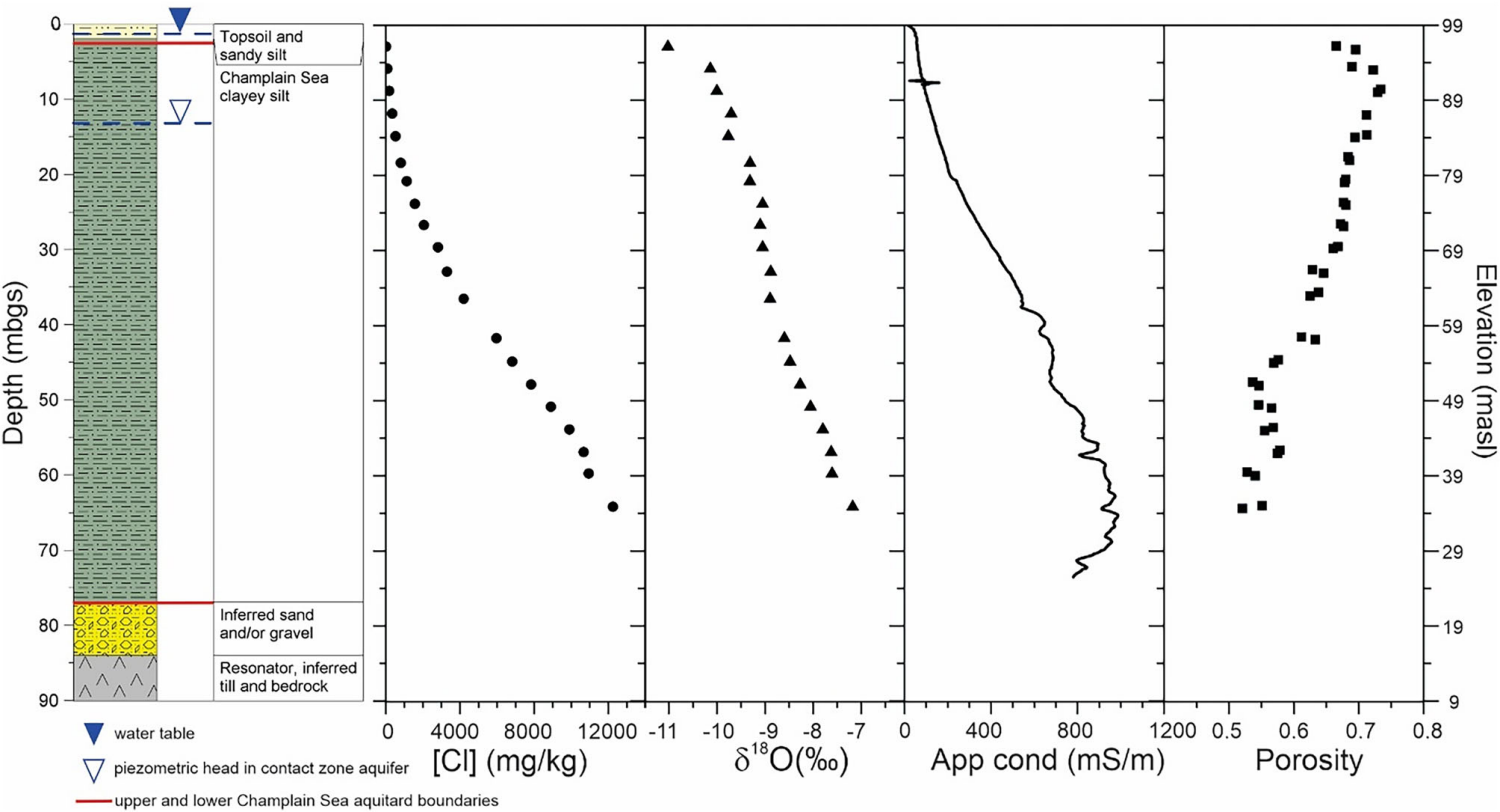
Porewater Cl concentrations, δ^18^O, borehole apparent conductivity log and porosity with depth.

At the borehole site (BH‐GSC‐BRK‐03), drilled in March 2014, microtremor recordings identified a hard resonator surface (till and/or bedrock) at ~84 m depth (15 masl; Figures 2 and 3; Crow et al. 2017). The aquifer elevation at 22.1 masl (76.9 m depth), inferred from groundwater modeling (see Section 4.2), is typically identified as gravel and/or sand in local water well records. The sand or gravel, sandy till, and uppermost bedrock are collectively known as the contact zone aquifer. A domestic water well, located ~500 m north of the borehole, is completed in the bedrock (WW2 in Figure S1). Its water well record documents a water level of 85.81 masl and a 7.6‐m thick sand and gravel unit overlying bedrock at a depth of 88.4 m.

Champlain Sea sediments in the Breckenridge borehole are characterized as clayey silt (i.e., mud) that occur from 2.0 to 76.9 m depth (97–22.1 masl) and are punctuated by approximately five fine sand seams (Crow et al. 2017). All cores and porewater samples for this study were collected from glaciomarine muds within the Champlain Sea sequence. A sandy silt layer (0.9–2.0 m depth, 98.1–97 masl) may result from deposition during the regression of the Champlain Sea, potentially representing more deltaic or littoral conditions as water depths were decreasing. Both the sandy silt and the overlying topsoil (0–0.9 m, 99–98.1 masl) likely behave as an unconfined surficial aquifer with predominantly lateral flow. The water table is expected to remain above 2.6 m depth because the shallow core at this depth does not show evidence of oxidation or fracturing.

The conceptual model of groundwater flow in the basin is largely controlled by its hydraulic connectivity (see Figure S1 and Section 1 of the Supporting Information). Precipitation infiltrates exposed bedrock upslope of the Champlain Sea plain to the northeast. Groundwater flows under the Champlain Sea sediments along the fault (defined by the Eardley Escarpment, Figure 2) and within the contact zone and/or bedrock aquifers, which effectively behave as one hydrostratigraphic unit because their permeability is orders of magnitude greater than that of the overlying Champlain Sea muds. Groundwater flow within the confined aquifer extends to the lower reaches of Breckenridge Creek where the mud thins or is absent, groundwater discharges, and the piezometric head approaches that of the stream water surface. Confined flow beneath the muds is expected to be relatively rapid because freshwater infiltration upslope displaced initial marine water. Further, nearby water wells are completed in the underlying contact zone aquifer and yield a potable domestic supply.

Precipitation exceeds evapotranspiration by more than 400 mm/a, which either infiltrates or generates surface runoff (Comeau et al. 2013). Most recharge into the surficial unit flows laterally to discharge into local streams and tributaries. The thin sandy silt overlying the Champlain Sea muds remains saturated from infiltrating precipitation and likely maintains the water table level close to ground surface. Therefore, the water table is higher than the piezometric surface in the underlying aquifers across most of the watershed, which drives groundwater flow vertically downward within the muds. However, the hydraulic conductivity of the unfractured Champlain Sea muds is so low, ranging from ~6E‐11 to 9E‐09 m/s (Hinton and Alpay 2020), that vertical groundwater flow through the muds represents a small fraction of the surficial recharge.

## Methods

The Geological Survey of Canada (GSC) collected geological, geophysical, geotechnical, and geochemical data using multidisciplinary methods in the Breckenridge watershed and borehole (Crow et al. 2017, 2020, 2021). The borehole was terminated at 75 m depth, ~9 m above till or bedrock; it was cased with PVC to a depth of 74.5 m to allow for borehole geophysical logging. A continuous apparent bulk conductivity log was collected along the borehole wall using an induction EM39 Conductivity Logger. For recovery of undisturbed sediment samples, an Osterberg hydraulic piston was used with 76‐mm diameter thin‐walled Shelby tubes at ~3 m intervals to a depth of 64.33 m. Twenty Shelby tube samples were collected and sealed on site to prevent moisture loss; they were stored upright at 5 °C. Cores were logged and sub‐sampled for various tests and treatments, including grain size, moisture content, shear strength, plastic and liquid limits (Crow et al. 2017). Within 1 month of sample collection, porewater was separated from the sediment samples by centrifuge (13,000 rpm for 30 min at 5 °C) followed by elemental analysis of porewaters by inductively coupled plasma optical emission spectrometry (ICP‐OES; [Cl] ±1.8%) at the GSC and isotopes by a Los Gatos Research‐Off‐Axis Integrated Cavity Output Spectroscopy laser system (LGR‐OA‐ICOS; δ^18^O ± 0.2‰ VSMOW) at the Environmental Isotope Laboratory, University of Waterloo, as described by Crow et al. (2017).

A simple hydrogeologic scenario of one‐dimensional vertical steady state flow and transient transport was modeled with finite‐element groundwater flow and transport software (FEFLOW, V7.5) and parameter optimization software (FePEST, V7.5). One‐dimensional flow is justified because the vertical hydraulic conductivity of the mud is orders of magnitude lower than that of the contact zone aquifer. Starting from the retreat of the Champlain Sea from the site, four model variables were adjusted to simulate vertical profiles of porewater Cl concentrations ([Cl]) and δ^18^O values together: vertical specific discharge (Darcy flux, *q*); longitudinal dispersivity, *α*_*L*_; initial Cl concentration ([Cl]_0_); and initial δ^18^O (δ^18^O_0_).

Using Darcy's law (Freeze and Cherry 1979),

(1) q=Ki=vn

*q* is directly controlled by varying the hydraulic conductivity (*K*) because the hydraulic gradient (*i*) is held constant in the model. In the one‐dimensional flow model, the vertical *q* is constant over the length of the sediment column; however, the average groundwater velocity, *v*, varies slightly and inversely with porosity (*n*). Thirty‐nine measurements of saturated gravimetric water content, along with three measurements of specific gravity (*G*_*s*_ = 2.76 ± 0.01), were used to determine *n* (Crow et al. 2017, 2021; Figure 3). The average porosity, *n* = 0.63, is typical for Champlain Sea muds (Tavakkoli et al. 2024) and is comparable to those determined independently using NMR methods (Crow et al. 2020). Porosity was interpolated for each model element and incorporated directly into the model.

Hydrodynamic dispersion, *D*_*L*_, is calculated according to:

(2) $$D_{L}=\alpha_{L}v+D_{e}$$

where *α*_*L*_ is the longitudinal dispersivity and *D*_*e*_ is the effective diffusion coefficient (Fetter 1992). In this study, *D*_*e*_ is held constant and *α*_*L*_ is optimized by FePEST.

The *D*_*e*_ of chloride, *D*_*e*_(Cl), was estimated as a function of the free‐water diffusion coefficient (*D*_0_) and *n*,

(3) $$D_{e}=D_{0}\cdot n^{m}$$

where *m* is an empirical constant (*m* = 2–2.5; Harrington et al. 2013) and *D*_0_ = 2.032E‐9 m^2^/s for Cl at 25 °C (Chemical Rubber Company 2010). As diffusion varies with temperature, the approach of Harrington et al. (2013, their equation 9) was used to calculate a temperature correction factor of 0.625 (from 25 °C to 8.6 °C, which is the average annual temperature at a depth of 19.4 m in a borehole 33 km to the east; unpublished data). Values of *m* = 2.3 and *n* = 0.63 provide a temperature‐corrected *D*_*e*_(Cl) = 4.422E‐10 m^2^/s, which was used for all simulations. The exception is Simulation 1, for which *D*_*e*_(Cl) was too high for an optimal solution; instead, *m* = 2.5 was used in Equation 2 to provide a lower *D*_*e*_(Cl) = 4.034E‐10 m^2^/s in Simulation 1. A comparison of using average porosity versus interpolated porosities for the calculation of *D*_*e*_ in Equation 3 showed that the former provides a better model fit with lower RMS (root mean square) of residual errors. The value of *D*_0_ for ^18^O in water was calculated directly for a temperature of 8.6 °C using Equation 1 in Easteal et al. (1984) and produces a *D*_*e*_(^18^O) = 5.050E‐10 m^2^/s using Equation 3. The ratio of the values, *D*_*e*_(^18^O)/D_e_(Cl) = 1.142, remains constant for all simulations. Parameter estimation in FePEST assumes that *α*_*L*_ for Cl and ^18^O are the same.

The modeling approach was to optimize *K*_*v*_, the vertical hydraulic conductivity (and therefore specific discharge, *q*, by Equation 1), and longitudinal dispersivity, *α*_*L*_, to match vertical profiles of both porewater [Cl] and δ^18^O values simultaneously. FePEST was used to determine the optimal values of *K*_*v*_ and *α*_*L*_ for each set of initial conditions. Initial conditions in the porewaters are unknown; therefore, the initial δ^18^O values were varied in a series of models for each fixed initial Cl concentration ([Cl]_0_) until the pair of initial conditions with the lowest weighted residual errors was achieved (see Section 5 of the Supporting Information and Figure S3). This procedure was repeated for seven possible [Cl]_0_ from 14,000 to 19,550 mg/kg, ranging from ~72 to 100% of seawater concentration during the Champlain Sea episode.

The model was designed as a two‐dimensional grid with boundary conditions representing one‐dimensional flow with a single column of quadrilateral elements and a discretization of 0.5 cm over a 90.06 m vertical domain (elevation *z* = 8.94–99 m). An initial hydraulic conductivity of 6E‐10 m/s was assigned to the muds based on simulations of the Kinburn site, located 21 km to the southwest in a thick sequence of Champlain Sea muds (Hinton and Alpay 2020); the value was subsequently optimized by FePEST. Without a surficial piezometer, an average water table depth of 1.3 m (97.7 masl) was estimated within the sandy silt sediment. The piezometric head of 85.81 m from the nearby domestic well was assigned to the contact zone aquifer (Figure 3). A fixed hydraulic gradient (*i* = 0.160) was maintained by assigning constant head boundary conditions at the specified upper (*h*_*c*_ = 97.7 m at *z* = 96.4 m) and lower boundaries (*h*_*c*_ = 85.81 m at *z* = 22.1 m) in all simulations (Figure 3). The range of possible hydraulic gradients was estimated as *i* = 0.08 to 0.24 or a ±50% error in *i*. The error range was obtained by varying the water table elevation from ground surface (*h*_*c*_ = 99 m) to the top of the upper boundary of unfractured Champlain Sea sediments (*h*_*c*_ = 96.4 m); the lower boundary was varied by ±5 m (*h*_*c*_ = 80.81–90.81 m). The model does not include effects of variable density flow or sediment consolidation.

**Figure 4:**
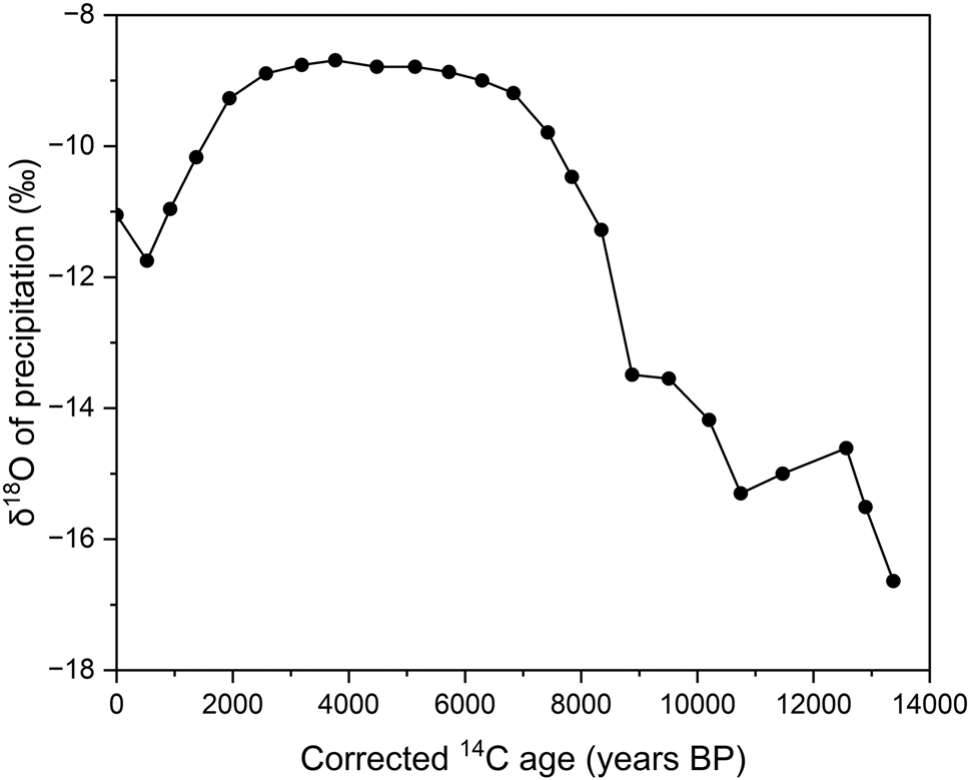
Precipitation δ^18^O as a function of time used for FEFLOW modeling (after Edwards et al. 1996).

The transient simulations of the model start at the estimated time of regression of the Champlain Sea, 10,406 calendar years BP, and end at the time of sampling (2014) for a model duration of 10,470 years. The estimated time that the Champlain Sea retreated from the site was based on the radiocarbon age of 10,100 ± 130 ^14^C years BP for a marine shell 16 km southeast of the Breckenridge borehole (Deschênes, GSC‐2189; Richard 1990). The radiocarbon age was corrected with CALIB software (v.8.2, calib.org) (Stuiver and Reimer 1993) with Δ*R* = 450 ± 400 years. The age compares well with an estimated age of marine regression of 10,340 years based on a glacio‐isostatic adjustment (GIA) model (unpublished analysis, Godbout et al. 2023). Transport boundary conditions were held steady for [Cl] (*C*(*t*) = 10 mg/kg at *z* = 96.4 m and *C*(*t*) = 500 mg/kg at *z* = 22.1 m). The upper boundary concentration was based on the uppermost porewater [Cl] result. Varying the Cl concentrations from 0 to 1000 mg/kg for the lower boundary condition had minimal impact on model results. Freeze and Cherry (1979) noted that water containing total dissolved solids of more than 2000–3000 mg/L is generally too saline to be potable; therefore, [Cl] is likely <1000 mg/kg. Transport boundary conditions for δ^18^O model inputs (at both *z* = 96.4 m and *z* = 22.1 m) use transient estimates of Holocene precipitation for Southern Ontario by Edwards et al. (1996) with corrections for ^14^C ages (CALIB software, v.8.2) at 500‐year intervals (Figure 4).

Automatic time‐step control was implemented in FEFLOW with a maximum time step of 50 years. FEFLOW and FePEST model settings are presented in greater detail in Sections 2 and 3 of the Supporting Information and Tables S1–S3. A modified version of this FEFLOW model for Cl only and without a freshwater lower boundary was compared with the results of an analytical 1D model (SUPER1D, Sudicky 1988) to ensure minimal numerical dispersion; the mean absolute residual error was <0.02%.

## Results

### Porewater Chemistry and Apparent Conductivity

**Figure 5:**
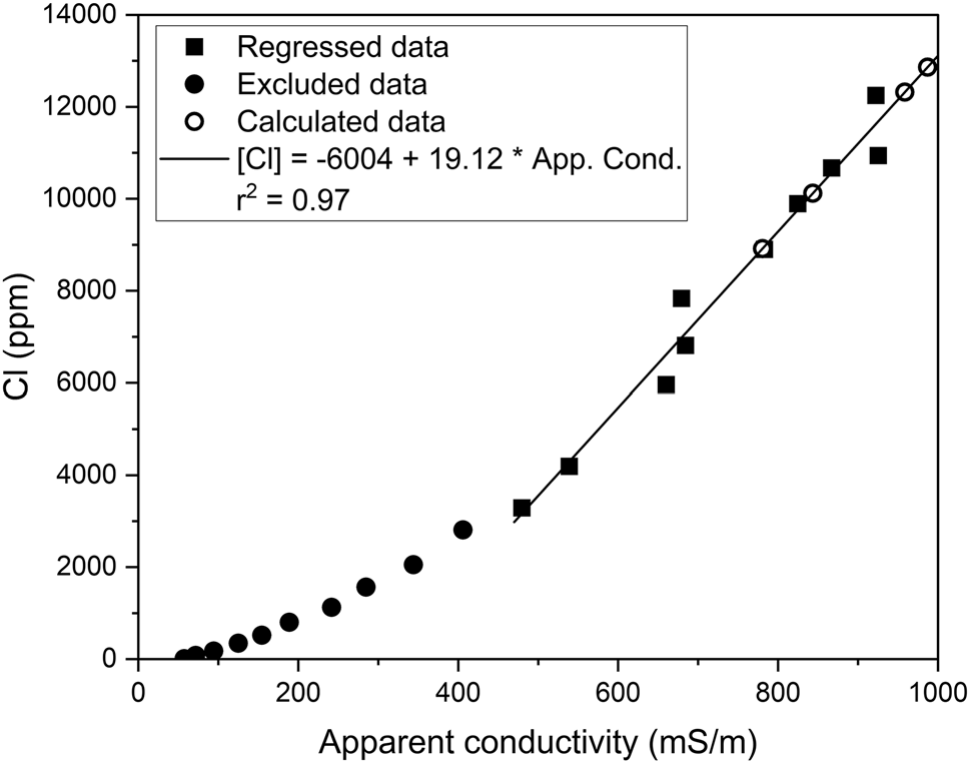
Relationship between borehole apparent conductivity and porewater chloride concentration.

The depth profile of [Cl] in porewater exhibits a pattern of increasing concentration with depth from a minimum of 9.6 mg/kg at 2.8 m depth to the peak measured value of 12,250 mg/kg in the deepest core sample at 64.1 m (Figure 3 and Table S5). Drilling conditions precluded core collection below this depth. However, the bulk apparent conductivity log extends deeper than the core samples and attains a peak at 65.2 m with a decrease of approximately 20% to the bottom of the profile at 73.5 m (Figure 3). The correlation between porewater [Cl] and bulk apparent conductivity (*r* ^2^ = 0.97) was used to calculate porewater [Cl] from the peak to the base of the borehole where there are no measured data (Figure 5 and Table S6). Only the highest values of apparent conductivity were included in the regression because apparent conductivity measures both solid phase and porewater conductivity in the formation. At higher porewater concentrations, apparent conductivity is dominated by porewater conductivity as the linear response demonstrates (Figure 5). The calculated Cl concentrations below 65 m depth are critical observation points to quantify the peak and decreasing porewater concentration profile; they also help define the location of the lower boundary of Champlain Sea sediments.

The shape of the δ^18^O depth profile differs notably from that of [Cl] (Figure 3). The changes in porewater δ^18^O are largest in the uppermost profile and reflect the changes in the isotopic content of precipitation over the last 2500 years (Figure 4), whereas the more gradual changes in [Cl] result from a constant input of dilute precipitation.

### Groundwater Modeling

To obtain a reasonable fit between measured and modeled [Cl] and δ^18^O profiles in the Breckenridge borehole, certain model conditions were essential: (i) downward flow; (ii) a lower freshwater boundary at ~77 m depth; and (iii) a plausible combination of [Cl]_0_ and δ^18^O_0_ values.

**Figure 6:**
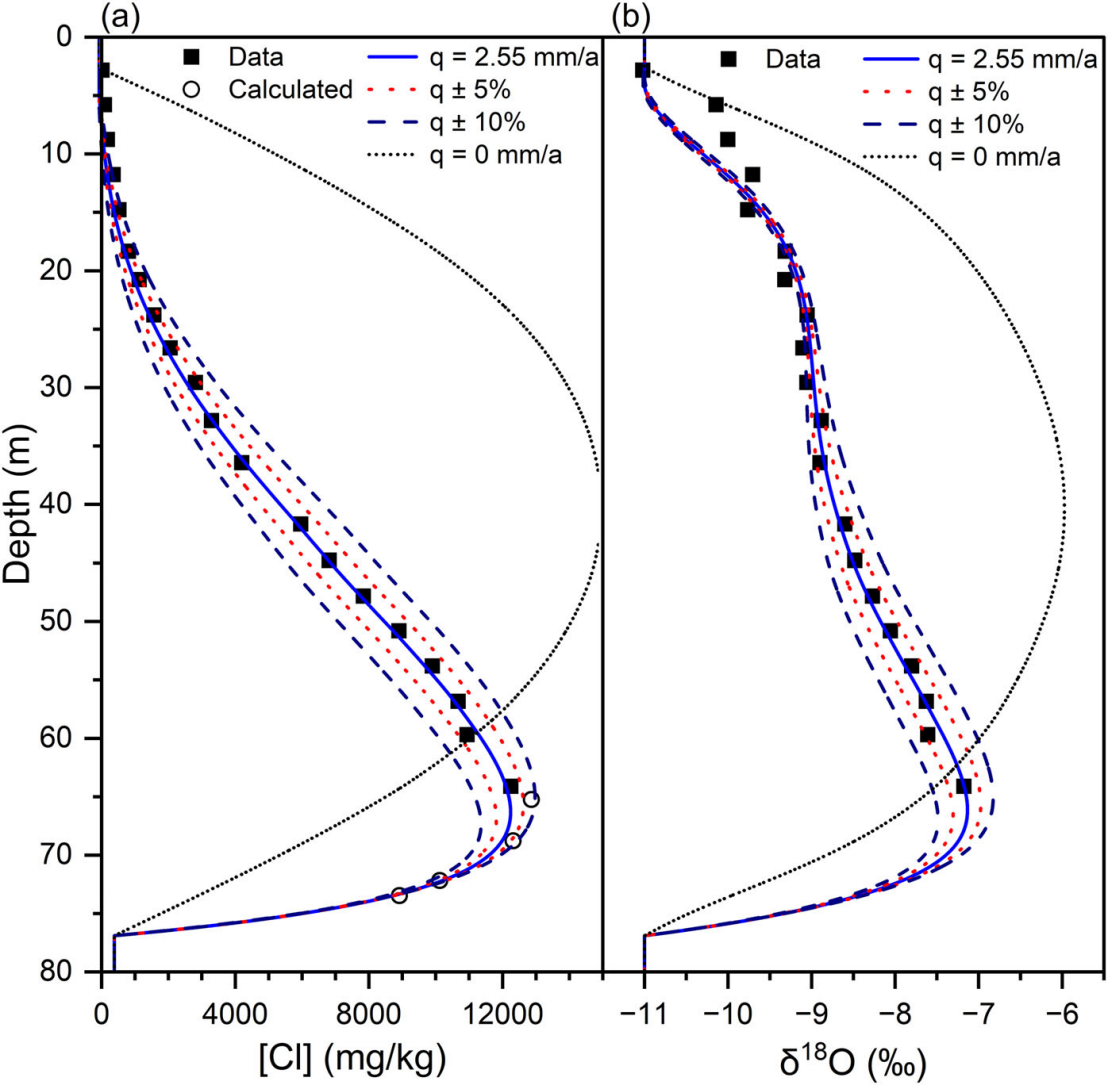
Simulation 5 ([Cl]_0_ = 16,000 mg/kg; δ^18^O_0_ = −5.49‰) showing the effect of a change in *q* of ±5% and ±10% and the comparison with a diffusion profile (*q* = 0 mm/a) for (a) Cl and (b) δ^18^O. Calculated [Cl] is estimated from apparent conductivity measurements in Figure 5.

Modeled [Cl] and δ^18^O profiles with depth indicate the influence of vertical groundwater flow (Figure 6). For this simulation, *q* = 2.55 mm/a and is well constrained; even a 5 or 10% change in *q* yielded a significant departure from the measured profile. If no groundwater flow is specified, both upward and downward diffusion of Cl towards the upper and lower aquitard boundaries produce a nearly symmetrical profile with the peak concentration at the middle depth and a concave downward shape for the upper profile. When downward flow is present, the concave upward shape of the measured values in the upper portion of the borehole is reproduced by the model (Figure 6).

**Figure 7:**
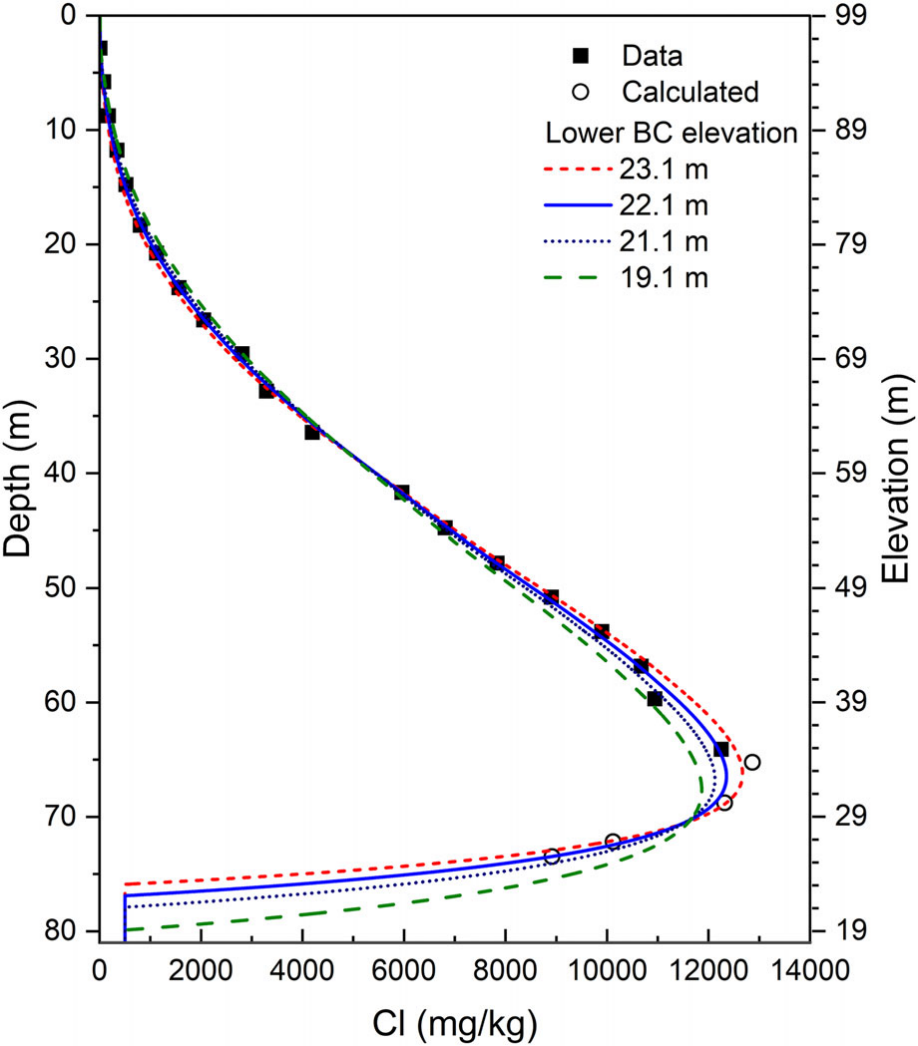
Simulations to determine the elevation of the lower boundary. Calculated [Cl] is estimated from apparent conductivity measurements in Figure 5.

The combination of downward advection and diffusion produces a steep decline in [Cl] from the peak to the lower boundary (Figure 6). The location of the lower boundary was determined by varying the elevation of the lower boundary (to the nearest 0.1 m) in simulations to find the position with the lowest weighted and unweighted RMS of residual errors. The simulations show that the calculated [Cl] narrowly constrains the location of the lower boundary (Figure 7). The optimal simulation has a contact zone aquifer elevation of 22.1 masl (Figure 7), which is ~7.1 m above the estimated depth of the resonator (till or bedrock) at 15 masl (Figure 2). The presence of the contact zone aquifer overlying bedrock is consistent with the nearby domestic water well record (WW2 in Figure S1). Glaciofluvial sand and gravel deposits or glaciolacustrine sandy varves overlying till and bedrock are also documented in the region (Johnston 1917; Gadd 1986; Cummings et al. 2011; Medioli et al. 2012).

**Figure 8:**
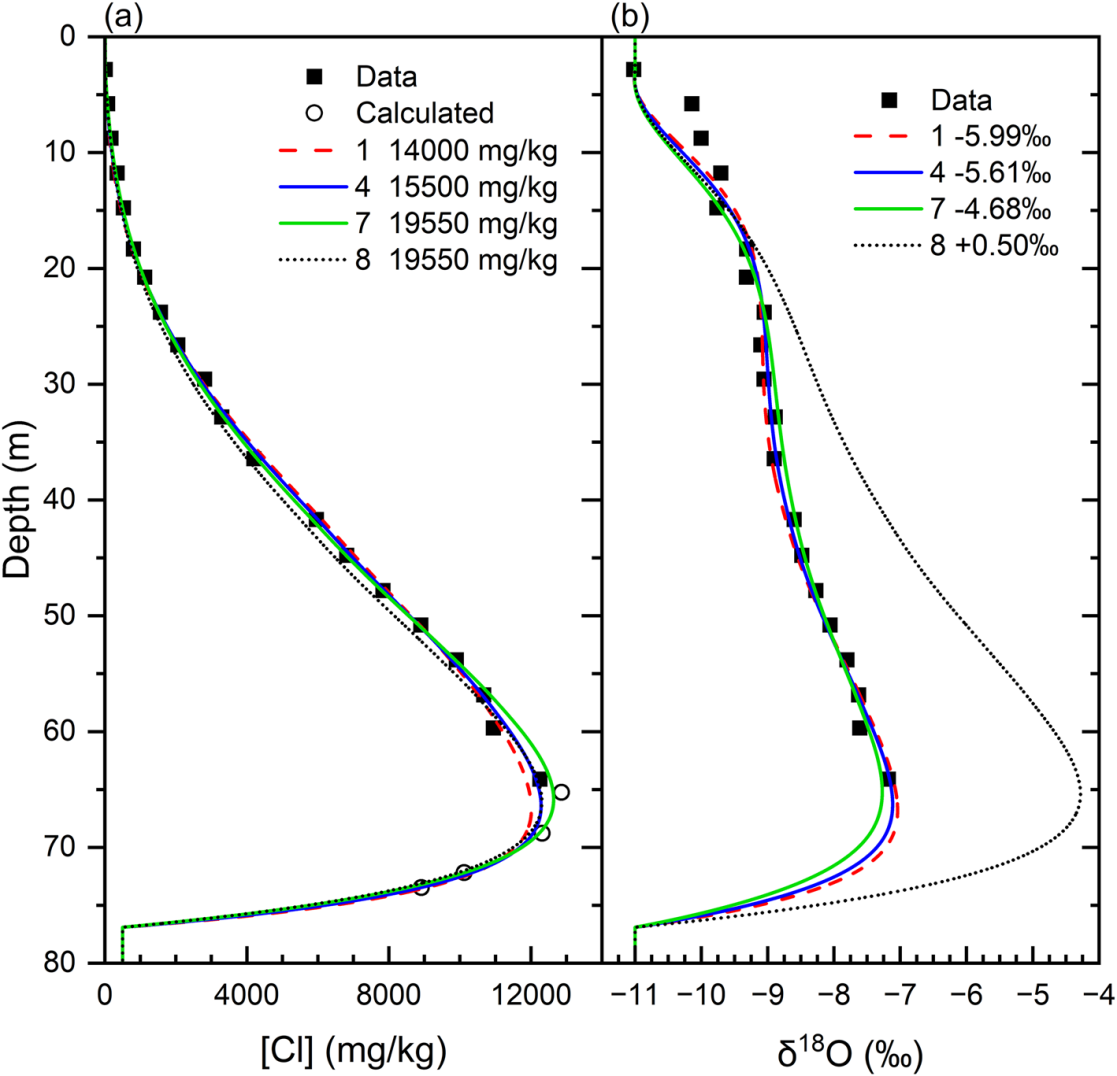
Model simulations for (a) Cl and (b) δ^18^O. Legend shows initial concentrations. Simulation results are found in Table 1. Calculated [Cl] is estimated from apparent conductivity measurements in Figure 5.

Various initial conditions of [Cl]_0_ and δ^18^O_0_ produce suitable matches between modeled and measured results (Simulations 1–7), each generating a different combination of *q* and *α*_*L*_ (Figure 8, Table 1). Simulation 8 does not reproduce the δ^18^O data (Figure 8), which demonstrates an unsuitable selection of δ^18^O_0_ for a given [Cl]_0_, in this case, the δ^18^O of 100% seawater. The simulations do not match the peak [Cl] concentrations for [Cl]_0_ ≤ 14,000 mg/kg, which was used as the lower limit of possible [Cl]_0_ (Figure 8). The [Cl] profile has greater control on q results than the δ^18^O profile, as shown by results of FePEST optimization of [Cl] and δ^18^O together, which consistently provide nearly identical *q* values to optimizations of [Cl] alone (see Section 5 of the Supporting Information and Table S4). Despite the wide range of [Cl]_0_ possible, there is a narrow range of *q*, from 2.40 to 2.79 mm/a.

**Table 1:** Simulation Results.

Sim	[Cl]_0_ mg/kg	δ^18^O_0_ ‰	*D*_*e*_ (Cl) 10^−10^ m^2^/s	*D*_*e*_ (^18^O) 10^−10^ m^2^/s	*K* 10^−10^ m/s	*q* mm/a	*α*_*L*_ m	RMS [Cl] mg/kg	RMS δ^18^O ‰ ^18^O
1^1^	14,000	−5.99	4.03	4.61	4.75	2.40	0.22	263	0.058
2^1^	14,500	−5.86	4.42	5.05	4.82	2.43	0.13	231	0.058
3^1^	15,000	−5.73	4.42	5.05	4.90	2.47	0.32	205	0.057
4^1^	15,500	−5.61	4.42	5.05	4.98	2.51	0.50	188	0.057
5	16,000	−5.49	4.42	5.05	5.05	2.55	0.67	177	0.052
6	18,000	−5.02	4.42	5.05	5.33	2.69	1.23	179	0.058
7	19,550	−4.68	4.42	5.05	5.53	2.79	1.58	202	0.26
8	19,550	0.50	4.42	5.05	5.67	2.86	1.58	305	1.54

^1^ Simulations with glacial meltwater end‐member δ^18^O between −22.4 and −30‰ (see Figure 9).

Abbreviations: RMS = Root mean square of unweighted residual errors; Sim = simulation.

In contrast to the wide range of [Cl]_0_, the range of δ^18^O_0_ that matches measured data is narrow, ranging from −5.99 to −4.68‰ (Table 1). Although all δ^18^O simulations match the overall shape of the profile, none of them closely match the shape of the curve between 5 and 15 m (Figure 8), which suggests that the δ^18^O precipitation data over the last 2000–3000 years from Southern Ontario may not be applicable for the Ottawa region (Figure 4).

Dispersivity, *α*_*L*_, varies from 0.13 to 1.58 m, with larger values for the higher initial concentrations (Table 1). Higher [Cl]_0_ requires larger *α*_*L*_ to reduce the greater stored mass of Cl, given that advection varies little among simulations. Although Simulations 1–7 fit the observed data, the RMS of residual errors is smallest for the initial conditions in Simulation 5 ([Cl]_0_ = 16,000 mg/kg) and increases with both increasing and decreasing values of [Cl] and δ^18^O (Table 1).

## Discussion

### Water Sources in the Champlain Sea

**Figure 9:**
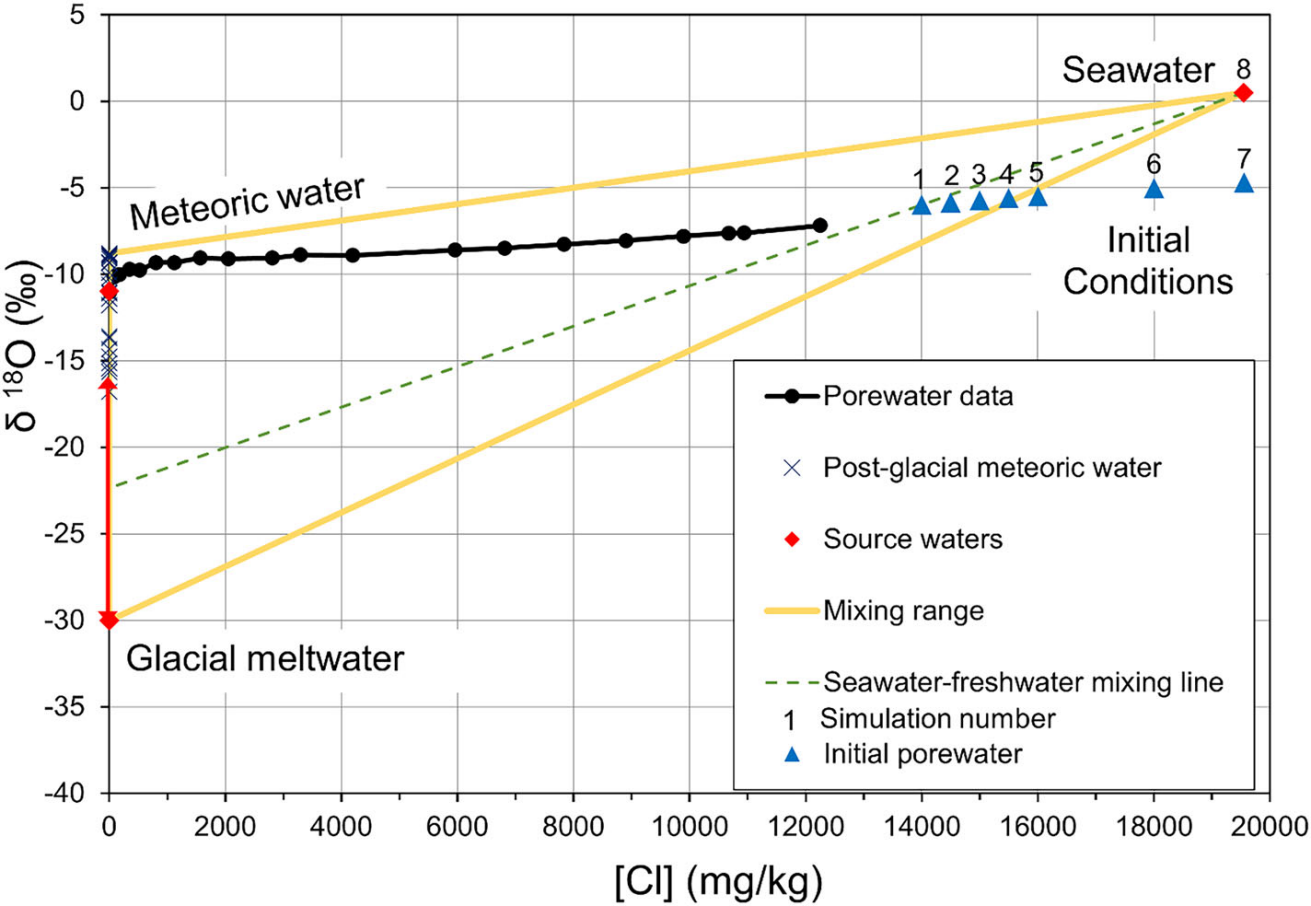
Water sources in the Champlain Sea. Numbers 1–8 indicate initial conditions for model simulations. The red line with arrows indicates the range of glacial meltwater δ^18^O values.

The composition of the Champlain Sea is a mixture of three water sources: seawater, glacial meltwater, and meteoric water. Each can be distinguished using Cl concentrations and δ^18^O values as tracers (Figure 9). Cl concentrations represent the proportion of seawater because both meltwater and meteoric water are freshwater sources with low solute content. Although measurements of two tracers allow quantification in a three‐component system (e.g., Hinton et al. 1994), the spatial and temporal variability of δ^18^O in both glacial meltwater and meteoric water makes quantitative three‐component separation uncertain. Nonetheless, the joint interpretation of [Cl] and δ^18^O is instructive in assessing the validity of the modeling results.

### Assessment of Groundwater Modeling Results

Despite using a simple conceptual model of 1D steady state flow and assumptions of uniform initial conditions and constant boundary conditions for [Cl], the agreement between the measured data and simulated results is remarkable as indicated, for example, by the means of absolute residual errors of 109 mg/kg for [Cl] and 0.13‰ for δ^18^O in Simulation 5 (Figure 6). The modeling results demonstrate that multiple pairs of [Cl]_0_ and δ^18^O_0_ can simulate the measured data using different q and α_L_ (Figure 8, Table 1). Identifying the best simulation is of relatively little importance for estimating q because its range (Table 1) is 0.39 mm/a (~15% of *q*), which is small in comparison to the magnitude of recharge to the surficial aquifer. Identifying the best simulation is pertinent for estimating diffusion and dispersion coefficients and for determining the initial porewater chlorinity of the Champlain Sea muds.

Although FePEST was used to optimize parameters *K* and *α*_*L*_, it also optimized *q* and *D*_*L*_ as evident in Equations 1 and 2 with fixed values of *i* and *D*_*e*_. However, comparison of the dispersivities and diffusion coefficients in Table 1 does not provide insight into which simulation is optimal because of the wide range and variability of *α*_*L*_ in the literature (Gelhar et al. 1992). Without measured values of *D*_*e*_ at the Breckenridge site, there are no simple means to select an optimal simulation. Even if a reliable *D*_*e*_ were available, different simulations could provide the required *D*_*L*_ for optimal solutions using different *α*_*L*_ values. For example, in additional simulations with *α*_*L*_ set to zero, the parameters *K* and *D*_*e*_ (i.e., = *D*_*L*_) were successfully optimized in FePEST.

A *D*_*e*_(Cl) of 2.0E‐10 m^2^/s was used for models in Champlain Sea muds by Quigley et al. (1983) and Desaulniers and Cherry (1989), assuming *v* = 0 mm/a. This value of *D*_*e*_(Cl) is 45–50% of the estimated *D*_*e*_(Cl) used in this study's simulations and 30–47% of the *D*_*L*_(Cl) (4.3E‐10 to 6.6E‐10 m^2^/s) because *v* ≠ 0. *D*_*L*_ obtained from porewater [Cl] profile modeling of the Kinburn site (Hinton and Alpay 2020) ranges from 3.9E‐10 to 4.5E‐10 m^2^/s, which is comparable but mostly lower than the range of values obtained in this study at the Breckenridge site.

Plotting the values of initial conditions in Figure 9 provides a means to assess whether the combinations of [Cl]_0_ and δ^18^O_0_ in Table 1 are realistic. It is important to recognize that the Champlain Sea was a water column stratified by salinity (Hillaire‐Marcel 1988); porewater incorporated into sediments during deposition was bottom water, which is denser and more saline than surface waters. Much of the overlying freshwater would have discharged as surface water to the ocean. It is a reasonable assumption that the component of glacial meltwater in the Champlain Sea represents thousands of years of accumulation from a large ice sheet area, an amount much larger than annual meteoric water input. Therefore, Champlain Sea waters would plot along a mixing line between the seawater and glacial meltwater end‐members (Figure 9). The seawater end‐member is well defined with a small decrease in δ^18^O value of 0.95 ± 0.09‰ from the Late Glacial Maximum to the Holocene (Adkins and Schrag 2001) with approximately half of the decrease occurring since the Champlain Sea episode (Adkins and Schrag 2003). However, the δ^18^O content of the glacial meltwater is poorly defined and may have changed as drainage areas and volumes to the Champlain Sea varied greatly, including contributions from glacial Lake Agassiz in central and western Canada (Katz et al. 2011). Person et al. (2007) review estimates for δ^18^O of meltwater from the Laurentide Ice Sheet with a range of −25 to −9‰, including three sites in glacial Lake Agassiz sediments with δ^18^O values of −24.5‰ in porewater (Remenda et al. 1994). Hillaire‐Marcel (1988) previously suggested a range of −30 to −16‰ but used the latter value for the glacial meltwater end‐member based on the isotopic content of biogenic carbonates. Adkins and Schrag (2001) used a mass balance calculation to suggest ice sheet δ^18^O globally was −29‰.

Modeling results should plot along possible mixing lines of seawater and glacial meltwater but several simulations do not represent plausible combinations of initial conditions. For example, a simulation with the [Cl]_0_ of 100% seawater should have δ^18^O_0_ contents of 0 to +0.5‰, yet the optimal model solution requires an unrealistic δ^18^O_0_ of −4.4‰ (Simulation 7, Figures 8 and 9). However, simulating a realistic combination of seawater [Cl]_0_ and δ^18^O_0_ does not necessarily match the measured data (Simulation 8, Figures 8 and 9). Assuming that the lowest glacial meltwater δ^18^O value is −30‰, then all initial conditions plotting below that glacial meltwater‐seawater mixing line are implausible (Simulations 5–7, Figure 9). Therefore, acceptable simulations (1–4) are limited to [Cl]_0_ ≤ approximately 15,700 mg/kg obtained by interpolation to the −30‰ mixing line (Figure 9).

### Champlain Sea Salinity of Bottom Water and Glacial Meltwater Mixing

The highest measured porewater [Cl] in the Breckenridge and Kinburn boreholes are 12,250 and 17,290 mg/kg, respectively, which represent a minimum salinity in Champlain Sea bottom water of 22.1 and 31.2 g/kg. Considering the modeled [Cl]_0_, then bottom water salinities may have been as high as 25.3–28.3 g/kg (14,000–15,700 mg/kg Cl) at Breckenridge and 31.8 g/kg (17,600 mg/kg Cl) at Kinburn (Hinton and Alpay 2020) for a significant duration of the Champlain Sea. In a two‐component mixing model between seawater and freshwater, they represent 74–80% and 90% seawater at the two sites, respectively.

The measured values of [Cl] and δ^18^O at a depth of 63.9 m at Breckenridge plot along a mixing line corresponding to the δ^18^O of freshwater of −20.1‰, which indicates that it could not have been more enriched. Simulation 1 plots along a mixing line with freshwater δ^18^O of −22.4‰, which represents the maximum δ^18^O of glacial meltwater (Figure 9). If freshwaters in the Champlain Sea were a mixture of meteoric water and glacial meltwater, then the glacial meltwater component would have been more depleted.

The maximum measured δ^18^O value of −7.18‰ at Breckenridge and the modeled δ^18^O_0_ values, ranging from −5.61 to −5.99‰ (Table 1), demonstrate the difficulties of interpreting δ^18^O signatures on their own to trace mixtures of glacial meltwater, seawater, and meteoric water in groundwater systems of the Champlain Sea basin. Initial mixtures of seawater and glacial meltwater that interact with groundwater in aquifers are less distinct in isotopic content. Therefore, analysis with other tracers (e.g., Figure 9) is necessary to interpret water sources.

### Hydrogeology of a Glaciomarine Aquitard

Champlain Sea sediments provide an example of a glaciomarine aquitard elevated above sea level and currently in a freshwater environment. At Breckenridge, Champlain Sea muds are composed mostly of silt (42–68%) and clay (28–57%) with minimal sand (0–4%) in the lower portion of the borehole (Crow et al. 2017). The model simulates vertical transport over the entire depth of the aquitard over more than 10^4^ years; therefore, the estimates of *q* allow for large scale estimation of *v* and *K*_*v*_. Using Simulations 1–4, the range of estimated *q* is narrow: 2.40–2.51 mm/a. Even when considering the range of porosity (*n* = 0.52 to 0.73), the full range of velocity is also narrow: 3.3–4.8 mm/a. The range of *K*_*v*_ for Simulations 1–4 in Table 1 is 4.75E‐10 to 4.98E‐10 m/s. Assuming the 50% uncertainty in the estimated hydraulic gradient (see Section 3), *K*_*v*_ could range from 2.8E‐10 to 8.8E‐10 m/s. Therefore, the simulation of porewater [Cl] and/or δ^18^O can be an effective approach to constrain the bulk *K*_*v*_ in glaciomarine muds.

Downward *q* at Breckenridge (2.40–2.51 mm/a) is 2.5–3 times larger than upward *q* (0.84–0.97 mm/a) for the Kinburn site (Hinton and Alpay 2020). However, the hydraulic gradient at Kinburn is approximately one third that at Breckenridge. Therefore, bulk *K*_*v*_ estimates at Breckenridge are comparable to values of 5.0E‐10 and 5.7E‐10 m/s at Kinburn. Flow estimates are also comparable to those from Benabdallah's (2010) 2D cross‐section model of Champlain Sea muds with vertical velocities of *v* = 0.5–2.8 mm/a (*q* = 0.3–1.7 mm/a) at four sites distal to a river.

The presence of aquifers underlying the Champlain Sea aquitard and their hydraulic connection to the ground surface are common features of the western Champlain Sea basin. Where contact zone and/or bedrock aquifers are recharged in outcrop zones, the aquifers provide a conduit for rapid freshwater flow beneath the muds and create a basal freshwater boundary. Most apparent conductivity borehole logs within Champlain Sea sediments in the Ottawa Valley either indicate: (i) near complete leaching of the original porewater salinity where sediment thickness is ~30 m or less, or (ii) the preservation of remnant marine salinity in areas similar to Breckenridge where sediments are thicker (Crow et al. 2015; see Section 4 of the Supporting Information and Figure S2). In thick sediments, apparent conductivity logs reach a maximum near the base of the Champlain Sea sequence with a steep gradient to underlying freshwater conditions in the contact zone aquifer (Crow et al. 2015). Only one of 30 apparent conductivity logs in the Ottawa Valley exhibits a symmetrical profile suggestive of diffusion‐dominant transport (Crow et al. 2015).

### Progression of Leaching

**Figure 10:**
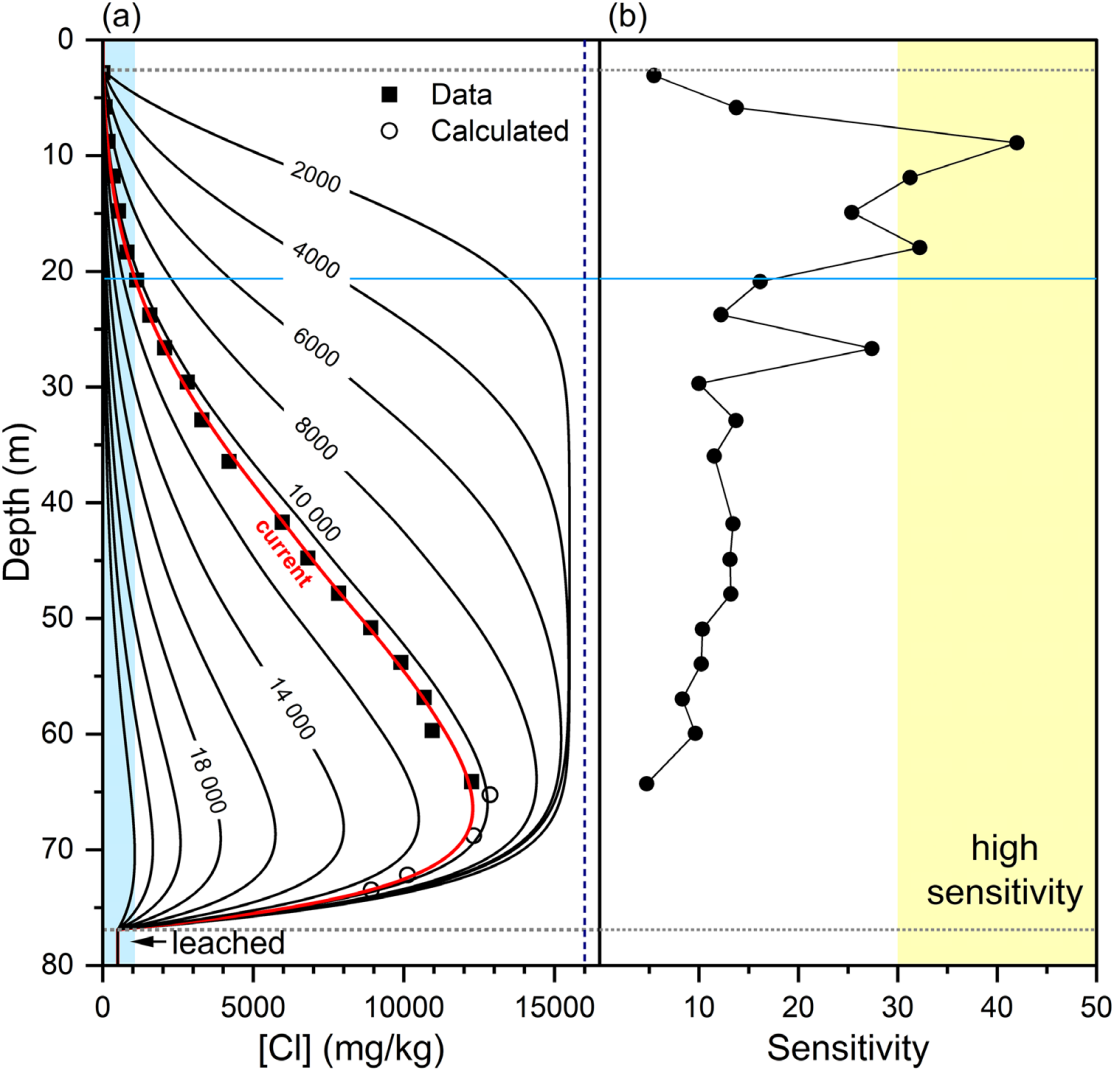
(a) Progression of leaching zone in years from model start. Data and solid red line correspond to model year 10,470. Blue shading indicates leached zone ([Cl] < 1072 mg/kg). (b) Sensitivity of core samples. Yellow zone indicates sensitivity >30. Dotted gray lines indicate upper and lower boundary conditions; horizontal blue line shows the modeled depth of leaching at the time of sampling.

The groundwater transport simulations provide an opportunity to quantify leaching in relation to the 2 g/L salinity threshold for the development of sensitivity (1072 mg/kg [Cl]). For example, Simulation 4 was extended to 24,000 years to investigate the past and future progression of leaching. Figure 10a shows the modeled porewater [Cl] profiles at 2000‐year intervals from the start of the simulation and also for sampling at year 10,470 (in 2014). Once the spatial extent of the influence of both the upper and lower boundary conditions overlap, in this case at ~8000 years, the peak concentration decreases rapidly. The blue zone (Figure 10a) indicates leached sediment based on the 2 g/L salinity criterion. The portion of each profile intersecting this zone indicates the downward advance of the leached zone over time. In the first 2000 years, the leached zone advanced 2.05 m below the upper boundary condition at a rate of 1.0 m/1000 years. Its rate of progression gradually increases with time; the current leached zone is at a depth of 20.63 m and is advancing at a rate of 2.5 m/1000 years. Its future progression is expected to reach a depth of 50.4 m at 20,000 years when the rate increases to 3.9 m/1000 years. The entire thickness of the aquitard is leached to the 2 g/L salinity criterion by model year 23,970 (15,514 AD). The progression of leaching is initially slower than the groundwater velocity (4.0 m/1000 years) because of upward diffusion and dispersion.

Measured geotechnical sensitivity in the Breckenridge core samples generally corresponds with the depth of leaching shown by the horizontal blue line in Figure 10. The three samples with high sensitivity occur within the leached zone. The lowest sensitivity samples occur where [Cl] is the highest at the base of the borehole and in the shallow zone where surficial processes such as cation exchange can also lower sensitivity. The sample at 26.7 m depth with a sensitivity of 27 may represent a zone in rapid transition; its salinity decreased from 7.3 g/L ([Cl] = 4100 mg/kg) at year 8000 to 3.7 g/L currently ([Cl] = 2070 mg/kg).

The aquitard muds are generally not permeable enough to supply domestic wells; therefore, water wells are usually completed in the underlying contact zone and bedrock aquifers. The presence of potable water supply across the region in these wells and the apparent conductivity logs for numerous boreholes across the Champlain Sea (Crow et al. 2015) support the conceptual model in which freshwater has flushed seawater salinity from most aquifers underlying the mud (see Supporting Information). The implication of a freshwater boundary at the base of the Champlain Sea muds is that diffusion also occurs at the lower boundary, halving the effective length of diffusion pathways. At the regional scale where the mud unit is thinner, the influence of overlapping upper and lower boundary effects occurred earlier, which caused more rapid leaching of porewater salinity. Furthermore, advection significantly increases solute transport (Figure 6) with the net result that advection, diffusion, and dispersion decreased the porewater salinity in most Champlain Sea muds. Preliminary generic modeling of the conceptual Champlain Sea setting suggests that transport processes are sufficient to have leached most sites with aquitard mud <30 m thick.

The understanding of groundwater flow has implications for porewater leaching and sensitivity development. Downward advection has a greater effect on the vertical advance of leaching than diffusion only. Without downward flow, leaching would not have progressed beyond a few meters (Figure 6). Similarly, at sites where groundwater flow is upward, a steep salinity gradient develops towards the surface and prevents leaching to greater depths, as shown at the Kinburn site (Hinton and Alpay 2020).

It is possible that the progression of leaching was a more significant factor for inhibiting retrogressive landslides in the past than more recently. Even in areas of thick Champlain Sea sediments with downward groundwater flow, such as Breckenridge, porewaters are currently leached to depths approaching that of stream incision. However, ~6500 years ago, leaching had progressed to a depth of 7.3 m, which would have limited the high sensitivity zone to shallower depths.

## Conclusions

One‐dimensional diffusion is reported to dominate solute transport at other sites in Champlain Sea muds (e.g., Quigley et al. 1983; Desaulniers and Cherry 1989). However, at the Breckenridge borehole, advection, dispersion, and diffusion contribute significantly to solute transport. A series of 1D groundwater transport models, using FEFLOW, simulated the depth profiles of measured [Cl] and δ^18^O in porewater simultaneously. In this study, modeling of [Cl] alone constrained *q* and *K*_*v*_ within a narrow range, particularly because the [Cl] profile captured both the peak value and decreasing concentrations towards the lower boundary. Simulation results show that specific discharges are small, *q* = 2.40–2.51 mm/a, and well constrained. The minimum initial [Cl] could be determined from [Cl] alone, in this case ~14,000 mg/kg or a salinity of about 25.3 g/kg.

With unknown initial conditions, there is no unique scenario to resolve. Therefore, numerous iterations and model optimizations were necessary, using FePEST, to identify combinations of initial conditions for both [Cl] and δ^18^O tracers that could simulate the measured profiles. Simultaneous matching of the two tracers does not limit the range of possible initial [Cl] of Champlain Sea bottom waters; however, it does constrain the range of initial δ^18^O from −5.99 to −4.58‰. Consideration of the three sources of Champlain Sea water, seawater, glacial meltwater, and meteoric water, allows further constraints to be placed on the initial paleowaters of the Champlain Sea at the Breckenridge site. Maximum initial chloride concentration and δ^18^O are ~15,700 mg/kg and −5.61‰, respectively, obtained using the three‐component mixing diagram and assuming a minimum glacial meltwater δ^18^O of −30‰. The mixing line between seawater and the simulation with minimum initial salinity establishes that the glacial meltwater component of bottom waters at the Breckenridge site has a maximum δ^18^O value of −22.4‰. Bottom waters are a mixture of between 72 and 80% seawater and 20 and 28% freshwater with δ^18^O ranging from −5.99 to −5.61‰. Without a seawater tracer such as Cl, δ^18^O alone is not a good tracer of the Champlain Sea because the mixture of seawater and glacial meltwater remains undefined.

Solute transport drives leaching of saline porewaters in the more than 10,000 years since the retreat of the Champlain Sea. This process of leaching marine salinity is a control on geotechnical sensitivity development in glaciomarine muds, which is a precursor condition for potential slope failures and retrogressive landslides. Leaching is a 3D process that occurs in concert with other factors leading to retrogressive landslides, including geomorphic evolution of the watershed through erosion, slope steepening, porewater pressure, and excessive loading (Lefebvre 1986).

Several aspects of the Breckenridge site provide a good opportunity to investigate the evolution of porewater chemistry. The thick sequence of Champlain Sea sediments preserves sufficient marine salinity in porewaters to interpret groundwater transport through the aquitard. The bulk apparent conductivity log supports calculation of porewater chlorinity and specific discharge at depths where porewater samples could not be collected. Freshwater at the base of the profile is observed in apparent conductivity logs of analogous sites in the Ottawa Valley (Crow et al. 2015). Therefore, the conceptual model of freshwater in the contact zone aquifer and 1D flow within the aquitard is viable.

More control on the initial salinity of Champlain Sea bottom waters would help narrow the scope and constrain the results of groundwater modeling. For example, integrated studies with paleoecological indicators can help constrain initial salinity. Similarly, empirical measurements of diffusion coefficients could further constrain modeling results. Leaching along hillslopes of eroding valleys where landslides initiate and propagate can be explored further by focusing porewater sampling across a valley transect and by coupling models of 2D or 3D groundwater transport with geomorphic evolution and evolving boundary conditions (Lefebvre 1986).

## Supporting information


**Data S1.** Supporting Information.

## Data Availability

The data that support the findings of this study are available in the Supporting Information of this article and in Crow et al. 2017.
