# Supplementary material for: Groundwater Transport in a Glaciomarine Aquitard: Paleosalinity and Landslide Implications
Source: Ground Water. 2026 Jan 13;64(1):49–63. doi: 10.1111/gwat.70045 (PMC12857530; doi:10.1111/gwat.70045)
Supplement: Supplementary file 1 — Data S1. Supporting Information. [file GWAT-64-49-s001.docx]

# Supporting information: Groundwater transport in a glaciomarine aquitard: paleosalinity and landslide implications

M.J. Hinton^1*^, S. Alpay^1^, and H.L. Crow^1^

^1^ Natural Resources Canada, Geological Survey of Canada, Ottawa, Ontario, Canada

*corresponding author at: Natural Resources Canada, Geological Survey of Canada, 601 Booth St., Ottawa, Ontario, Canada, K1A 0E8, [marc.hinton@nrcan-rncan.gc.ca](mailto:marc.hinton@nrcan-rncan.gc.ca%20)

Email addresses:

M.J. Hinton: [marc.hinton@nrcan-rncan.gc.ca](mailto:marc.hinton@nrcan-rncan.gc.ca)

S. Alpay: [sam.alpay@nrcan-rncan.gc.ca](mailto:sam.alpay@nrcan-rncan.gc.ca%20)

H.L. Crow: [heather.crow@nrcan-rncan.gc.ca](mailto:heather.crow@nrcan-rncan.gc.ca%20)

Number of pages: 11

Number of tables: 6

Number of figures: 3

Section 1. Study site cross-section

Additional site information from lidar topographic information and water well records, combined with the HVSR results (Figure 2), support the conceptual model for the site. Figure S1 presents a map view and composite cross-section of the Breckenridge watershed, which include the study borehole (BH-GSC-BRK-03), six water well records and bedrock geological features. Panel A shows the topography for the area with 1 m contours derived from lidar surveys ([Forêt ouverte](https://www.foretouverte.gouv.qc.ca/) website, Quebec Ministry of Natural Resources and Forests). Water well records were obtained from the Système d'information hydrogéologique website ([Système d'information hydrogéologique (SIH)](https://www.environnement.gouv.qc.ca/eau/souterraines/sih/index.htm)) of the Quebec Ministry of the Environment, the Fight against Climate Change, Wildlife and Parks. The Système d'information géominière website ([SIGÉOM | Système d'information géominière | Interactive map](https://sigeom.mines.gouv.qc.ca/signet/classes/I1108_afchCarteIntr?l=A)) of the of the Quebec Ministry of Natural Resources and Forests provided the locations of bedrock outcrop and the unconformity contact. Panel B shows the stratigraphic cross-section based on the borehole log of this study at BRK-03, water well record logs and the HVSR survey. The upland portion above the Eardley Escarpment (Eardley fault) to the northeast is Precambrian bedrock. Beneath the Champlain Sea plain, bedrock includes both Ordovician dolostones and limestones in the south and southeastern portions of the watershed and Precambrian bedrock below the north and northeastern portions as indicated by the unconformity contact (Panel A). The topographic rise immediately to the south of lower Breckenridge Creek is a ridge of Ordovician bedrock. A layer of gravel and/or sand, up to approximately 8 m thick, overlies the bedrock and along with the upper bedrock form the contact zone aquifer. This aquifer is confined by the muds of the Champlain Sea aquitard that form a nearly planar upper surface dissected by stream and fluvial erosion, particularly in the lower reaches of Breckenridge Creek.

Expected groundwater flow paths are indicated in Figure S1B with blue arrows. Groundwater flow in the contact zone aquifer originates as recharge in the Precambrian upland area with flow along the Eardley fault. Groundwater then flows in the contact zone aquifer beneath the mud and likely discharges into the lower reaches of Breckenridge Creek where the mud is thin or potentially absent, and ultimately to the Ottawa River to the west. The potentiometric surface in the contact zone aquifer is indicated by the water levels in the water wells. It is highest near the Eardley Escarpment and generally decreases down basin where it is likely near the surface water elevation of the creek. The water table is close to ground surface in either surficial sand, sandy silt or within the uppermost mud which sets up a vertical hydraulic gradient through the mud, thereby justifying the assumption of one-dimensional vertical groundwater flow within the aquitard.

Section 2. FEFLOW model settings

FEFLOW model settings are summarized in Table S1. Although the model is set up as a two-dimensional vertical model, there is only one column of rectangular elements with identical boundary conditions on each side of the elements that effectively renders a one-dimensional simulation. The vertical height of each element is 0.5 cm.

Section 3. FePEST model settings

FePEST was used to determine the optimal values of K_v_ and α_L_ for each set of initial conditions ([Cl]_0_ and δ^18^O_0_). Initial conditions in the porewaters are unknown; therefore, the initial δ^18^O values were varied systematically in a series of models for each fixed initial Cl concentration ([Cl]_0_) until the pair of initial conditions with the lowest objective function (Φ, sum of weighted residual errors squared) were achieved (Section 5). The process was repeated for each [Cl]_0_. The default FePEST model settings for optimization control and regularization were accepted, and parameter selections are presented in Table S2. Parameters are set to be constant for all saturated, unfractured Champlain Sea mud elements (z = 22.1-96.4 m).

The weighting of observations is particularly important for optimizations. Since Cl concentrations for observation points vary over several orders of magnitude, they require a weighting calculated from the inverse measurement noise to ensure that observations with small values are not under-represented and those of lower confidence are reduced (Table S3). Confidence intervals for observation points were specified in FEFLOW as the square root of the concentrations. The weighting for δ^18^O observations was obtained by trial and error with intent to equalize the overall contribution of each observation group to the total objective function (Table S3).

Figure S1. Breckenridge area topographic map, composite cross-section, study site borehole and water wells. Panel A shows topography with 1-m contour intervals from a lidar survey. Numbered or dated shaded areas are landslide scars mapped by Brooks et al. (2021). Panel B shows surface and resonator cross-sections along with borehole and projected water well logs. Arrows show conceptual groundwater flowpaths.


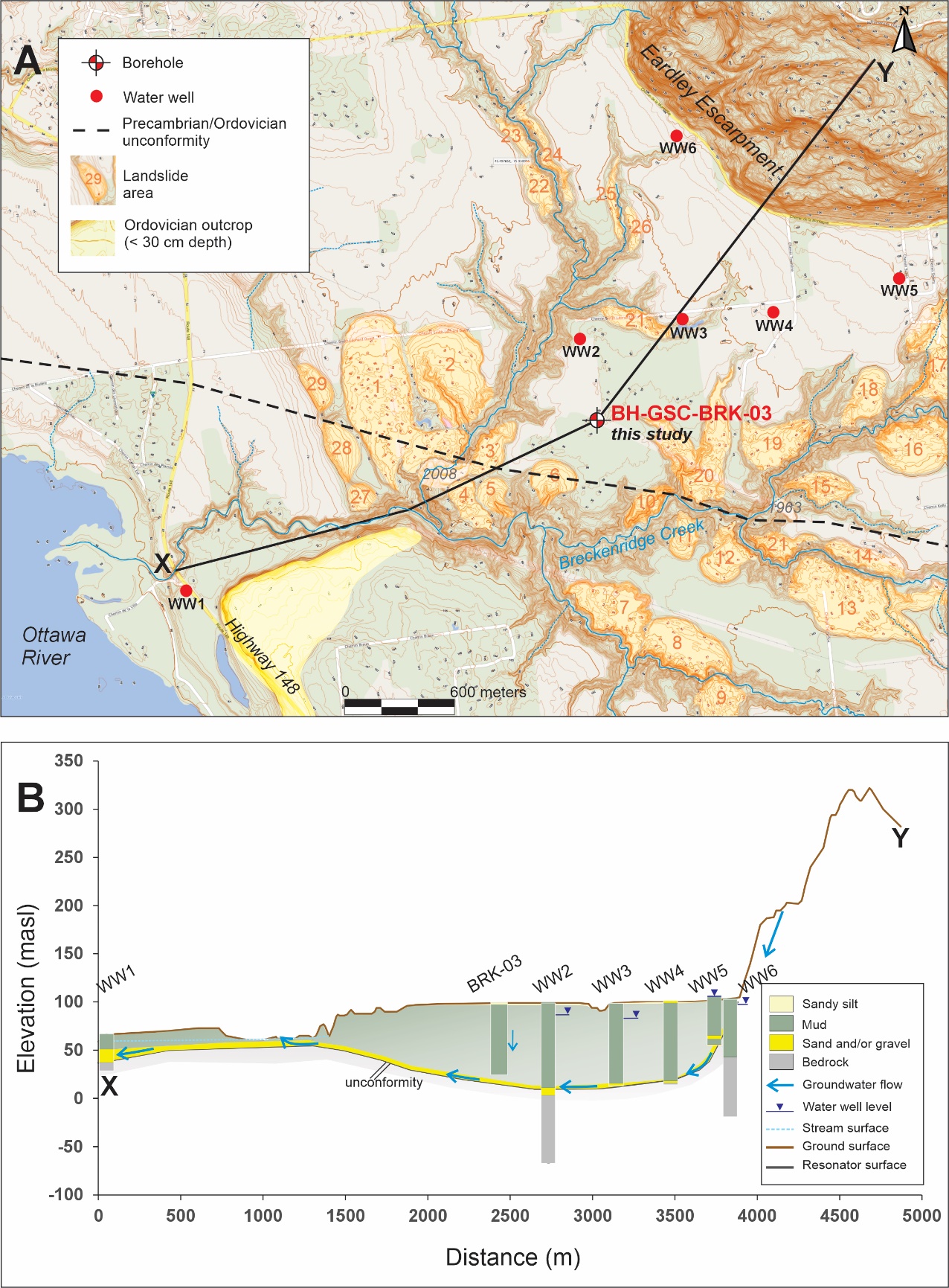


**Table S1**

**FEFLOW model settings**

| **Property** | **Description/value** |
| --- | --- |
| Projection | Vertical, planar |
| Simulation | Standard (saturated) groundwater-flow equation |
| Fluid flow | Steady state |
| Transport | Transient |
| Dimensions | 2 |
| Nodes per element | 4 |
| Element type | Quadrangle |
| Number of mesh elements | 18012 |
| Number of mesh nodes | 36026 |
| Domain width | 0.1 m |
| Domain height | 90.06 m |
| Time step control | Automatic, Adams-Bashforth/Trapezoid rule (AB/TR) predictor-corrector |
| Initial time step length | 1e-6 [a] |
| Final simulation time | 10470 [a] |
| Time step growth factor | Unrestricted |
| Max. time step | 50 [a] |
| Error tolerance | 0.1 [*10^-3^], Euclidean L2 integral (RMS) norm |
| Transport equation | Convective form |
| Fluid viscosity | Constant |
| Fluid density | Constant |
| Equation solver | Direct solver, PARADISO |

**Table S2**

**FePEST parameter settings**

| **Parameter** | **Transform** |
| --- | --- |
| Conductivity (K_v_) | log |
| Longitudinal dispersivity, α_L_(Cl) | none |
| Longitudinal dispersivity, α_L_(^18^O) | tied (equal) to α_L_(Cl) |

**Table S3**

**FePEST observation definition settings**

| **Observation** | **Weight** |
| --- | --- |
| Mass concentration - Cl | Inverse of measurement noise, δ 10 |
| Mass concentration - ^18^O | 7 |

Section 4. Apparent conductivity profiles of Ottawa Valley boreholes in Champlain Sea sediments

Geophysical logs provide continuous data that highlight in situ changes in properties, which may not be visible in the core. Apparent conductivity logs collected using a Geonics Ltd. EM39 probe provide a measure of bulk sediment conductivity, which responds to both mineral grains and porewater. In fresh porewater conditions, the tool identifies lithological changes associated with variations in mineralogy, grain size, and to a lesser extent, porosity. Geological Survey of Canada experience in Champlain Sea sediments of the Ottawa Valley has shown that, in the presence of conductive pore fluids, the tool response follows the trends of the pore fluid conductivity (Figure 3).

Six examples of apparent conductivity logs are shown from across the Ottawa Valley (Figure S2) in which peak mud conductivity ranges from 85 mS/m (relatively fresh porewater) to 987 mS/m (Breckenridge, 66% remnant sea water salinity). These examples display an asymmetrical curve in which the peak conductivity exists near the base of the muds as a result of both downward groundwater flow and diffusion. This contrasts with a diffusion-only scenario where the conductivity peak would be more centrally located within the mud (e.g., Figure 6, q = 0 mm/a). The lower conductivity at the top and bottom of the profiles reflects fresh water in the surficial and contact zone aquifers which results in both upward and downward diffusion of seawater ions from the peak values.

Figure S2. Six apparent conductivity logs are shown across the Ottawa Valley (data from Crow et al., 2015). Conductivity values represent a range of pore fluid conductivities with solute transport by downward flow and diffusion.


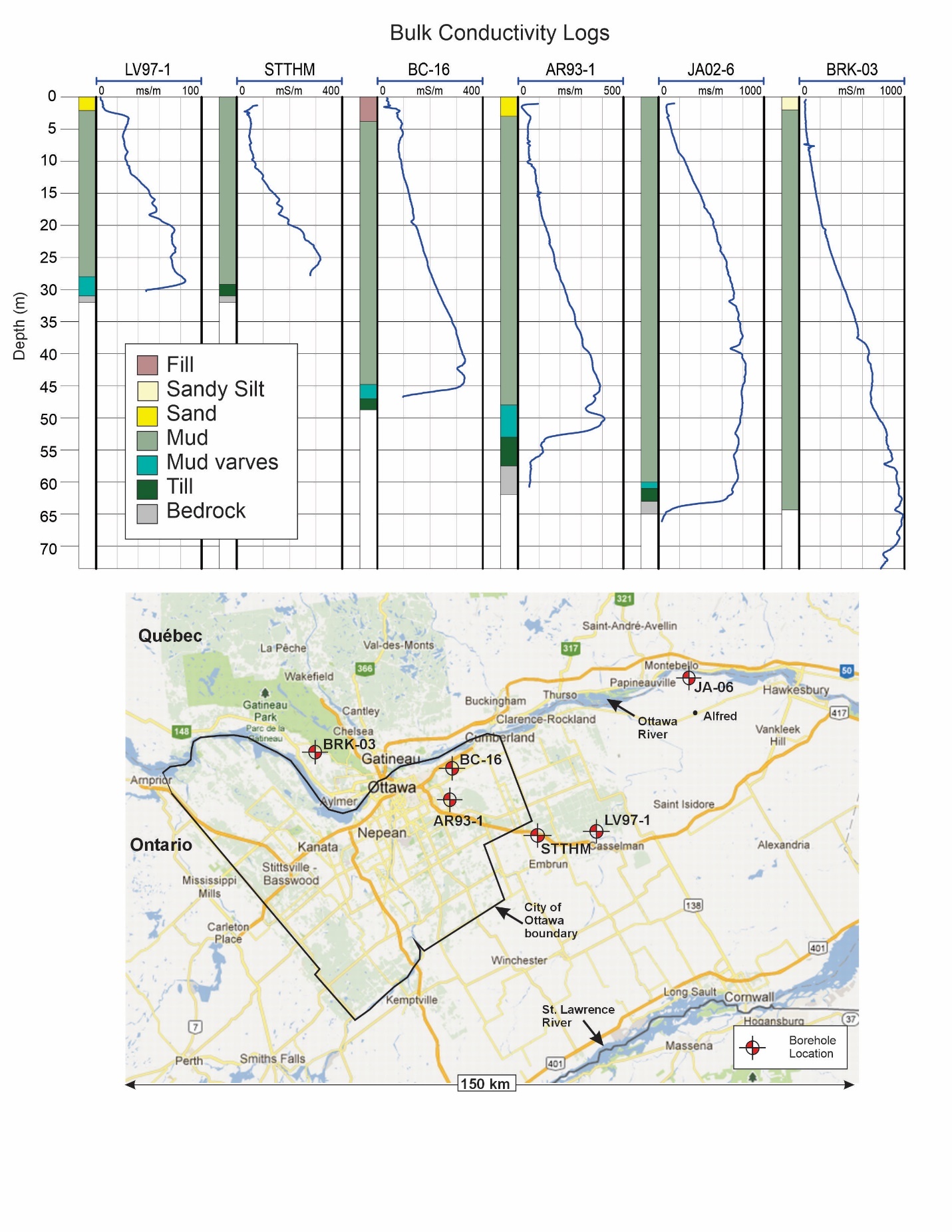


Section 5. Results

An example is provided to show how FePEST was used to determine the optimal combination of [Cl]_0_ and δ^18^O_0_. As part of simulation 5 (Table 1 and Figures 6 and 8), the FePEST model was run several times with [Cl]_0_ = 16000 mg/kg using different values of δ^18^O_0_. Figure S3 shows the component (Cl and ^18^O) and total objective functions for each FePEST model optimization. The model with the minimum total objective function (δ^18^O_0_ = -5.49‰) was considered the optimal combination of [Cl]_0_ and δ^18^O_0_ for the specified [Cl]_0_, in this case 16000 mg/kg, and is reported in Table 1. The process was repeated for each [Cl]_0_ (i.e., each simulation in Table 1). For simulation 8, only the combination of [Cl]_0_ = 16000 mg/kg and δ^18^O_0_ = 0.50‰ was optimized using FePEST with the sole intent to demonstrate that seawater composition of initial porewater does not provide a viable solution. Of note is that all optimizations resulted in very similar K_v_ values and, therefore, q estimates; the resulting q for all the model optimizations in Figure S3 differed by less than 0.005 mm/a.

Figure S3. Example of minimizing the objective function to find the optimal δ^18^O_0_ for [Cl]_0_ = 16000 mg/kg.


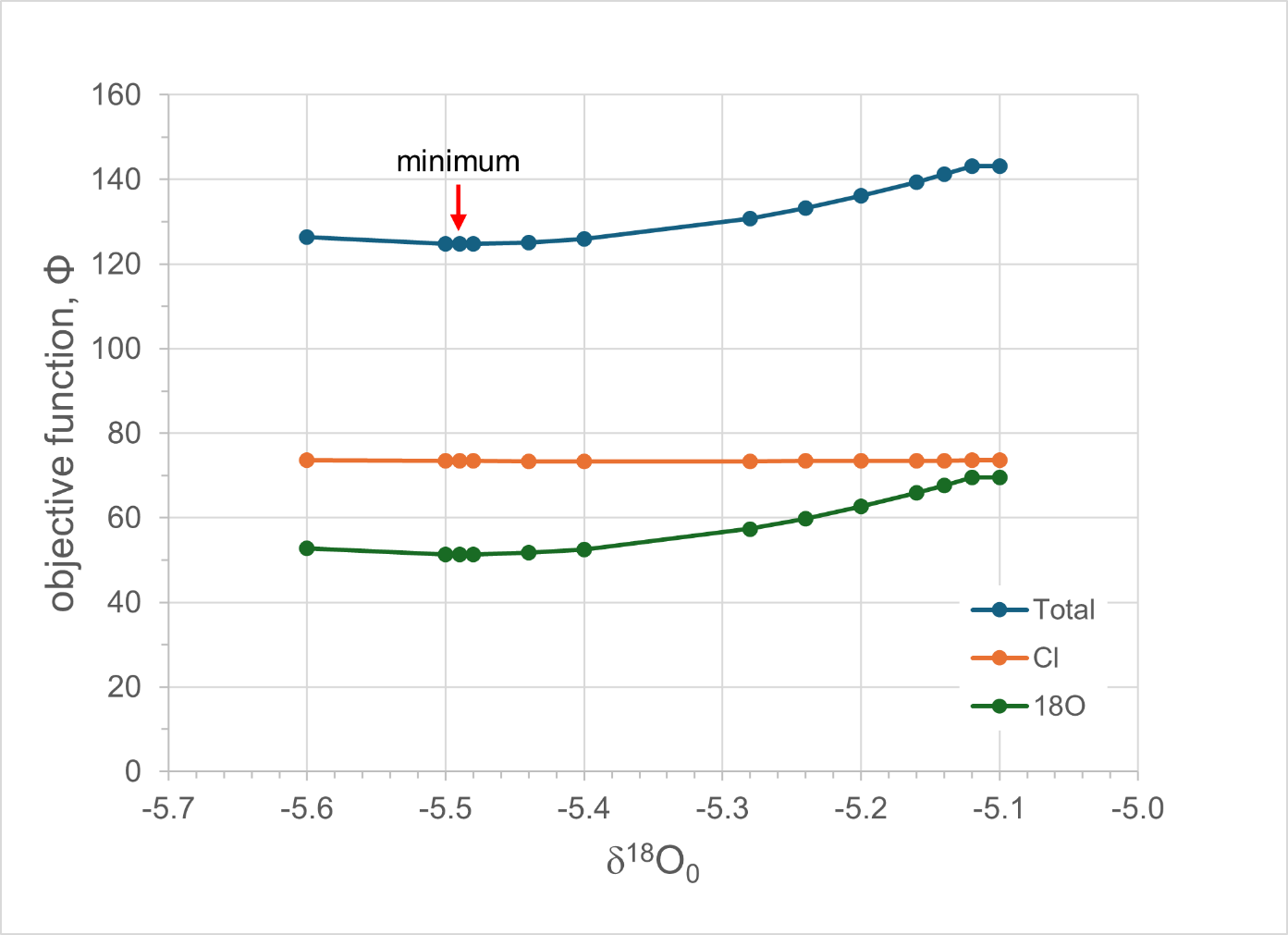


Simulations 1-7 were repeated using i) only the [Cl] data (simulations 1-Cl to 7-Cl) and ii) only the δ^18^O data (simulations 1-^18^O to 7-^18^O) for comparison with the joint simulations. Simulations using only [Cl] data provide nearly identical q results to those of joint simulations of [Cl] and δ^18^O (Table S4); simulations using only δ^18^O data range from 0.2% to 2.7% lower than joint simulations, which demonstrates that each independent dataset can provide accurate q estimates. Matching the temporal variations in the isotopic precipitation data proves sufficient to estimate q using porewater isotopic data alone despite the absence of measurements below the peak chloride concentration.

**Table S4**

**Comparison of results from joint [Cl] and δ^18^O simulations**

|  |  | **[Cl] + δ^18^O** | |  | **[Cl] only** | |  | **δ^18^O only** | |
| --- | --- | --- | --- | --- | --- | --- | --- | --- | --- |
| **[Cl]_0_** | **δ^18^O_0_** | **sim** | **q** |  | **sim** | **q** |  | **sim** | **q** |
| **mg/kg** | **‰** |  | **mm/a** |  |  | **mm/a** |  |  | **mm/a** |
| 14000 | -5.99 | 1 | 2.40 |  | 1-Cl | 2.40 |  | 1-^18^O | 2.33 |
| 14500 | -5.86 | 2 | 2.43 |  | 2-Cl | 2.44 |  | 2-^18^O | 2.38 |
| 15000 | -5.73 | 3 | 2.47 |  | 3-Cl | 2.47 |  | 3-^18^O | 2.43 |
| 15500 | -5.61 | 4 | 2.51 |  | 4-Cl | 2.51 |  | 4-^18^O | 2.47 |
| 16000 | -5.49 | 5 | 2.55 |  | 5-Cl | 2.55 |  | 5-^18^O | 2.52 |
| 18000 | -5.02 | 6 | 2.69 |  | 6-Cl | 2.69 |  | 6-^18^O | 2.68 |
| 19550 | -4.68 | 7 | 2.79 |  | 7-Cl | 2.79 |  | 7-^18^O | 2.79 |

sim = simulation

**Supporting data**

Supporting data are provided in Tables S5 and S6. Table S5 includes additional porewater analyses (Br, Na, Ca and δ^2^H). Table S6 reports the calculated [Cl] determined from the correlation with apparent conductivity (Figure 5). Water content, void ratio, porosity, undisturbed and remolded shear strengths and sensitivity data are reported in Crow et al. (2017).

**Table S5**

**Porewater concentrations and isotopic compositions.**

| **Core** | **Depth** | **Elevation** | **Cl** | **Br** | **Na** | **Ca** | **^18^O** | **^2^H** |
| --- | --- | --- | --- | --- | --- | --- | --- | --- |
|  | **m** | **masl** | **mg/kg** | **mg/kg** | **mg/kg** | **mg/kg** | **‰ (VSMOW)** | **‰ (VSMOW)** |
| 3 | 2.83 | 96.17 | 9.6 | 0.10 | 21.9 | 20.07 | -11.02 | -76.34 |
| 4 | 5.78 | 93.22 | 80.5 | 0.32 | 170 | 17.708 | -10.14 | -70.09 |
| 5 | 8.75 | 90.25 | 174 | 0.78 | 276 | 10.455 | -10.00 | -68.06 |
| 6 | 11.76 | 87.24 | 345 | 1.39 | 436 | 8.352 | -9.71 | -64.82 |
| 7 | 14.77 | 84.23 | 520 | 1.98 | 577 | 9.38 | -9.76 | -65.28 |
| 8 | 18.32 | 80.68 | 802 | 2.92 | 758 | 12.1 | -9.32 | -62.74 |
| 9 | 20.76 | 78.24 | 1124 | 4.08 | 998 | 16.95 | -9.32 | -62.39 |
| 10 | 23.78 | 75.22 | 1565 | 5.41 | 1284 | 23.68 | -9.05 | -61.57 |
| 11 | 26.60 | 72.40 | 2053 | 7.24 | 1648 | 34.18 | -9.10 | -61.83 |
| 12 | 29.56 | 69.44 | 2810 | 9.50 | 1958 | 41.64 | -9.06 | -61.43 |
| 13 | 32.83 | 66.17 | 3290 | 11.50 | 2435 | 41.7 | -8.88 | -60.39 |
| 14 | 36.43 | 62.57 | 4194 | 14.30 | 2936 | 53.2 | -8.90 | -60.77 |
| 15 | 41.68 | 57.32 | 5958 | 19.70 | 4002 | 82.5 | -8.60 | -58.99 |
| 16 | 44.78 | 54.22 | 6811 | 23.60 | 4458 | 89.3 | -8.48 | -58.17 |
| 17 | 47.82 | 51.18 | 7833 | 26.30 | 4939 | 88.1 | -8.27 | -57.35 |
| 18 | 50.79 | 48.21 | 8900 | 30.50 | 5415 | 103.9 | -8.06 | -56.12 |
| 19 | 53.80 | 45.20 | 9896 | 34.60 | 6071 | 109.7 | -7.80 | -54.56 |
| 20 | 56.83 | 42.17 | 10672 | 36.20 | 6317 | 113.5 | -7.63 | -53.44 |
| 21 | 59.68 | 39.32 | 10940 | 38.20 | 6405 | 92.6 | -7.61 | -53.23 |
| 23 | 64.10 | 34.90 | 12249 | 40.60 | 7208 | 55.8 | -7.18 | -50.46 |

**Table S6.**

**Calculated porewater chloride concentrations from apparent
conductivity logs (Figure 5).**

| **ID** | **Depth** | **Elevation** | **Apparent conductivity** | **Calculated Cl concentration** |
| --- | --- | --- | --- | --- |
|  | **m** | **masl** | **mS/cm** | **mg/kg** |
| 24C | 65.24 | 33.76 | 986.95 | 12865 |
| 25C | 68.78 | 30.22 | 958.43 | 12319 |
| 26C | 72.18 | 26.82 | 843.40 | 10120 |
| 27C | 73.48 | 25.52 | 780.49 | 8917 |

**References**

Brooks, G.R., Medioli, B.E., Aylsworth, J.M. and Lawrence, D.E., 2021. An updated compilation of radiocarbon dates relating to the age of sensitive-clay landslides in the Ottawa Valley, Ontario-Quebec. Geological Survey of Canada, Open File 7432, 98 pp. <https://doi.org/10.4095/327843>.

Crow, H., Good, R.L., Hunter, J.A., Burns, R.A., Reman, A. and Russell, H.A.J., 2015. Borehole geophysical logs in unconsolidated sediments across Canada. Geological Survey of Canada, Open File 7591, 39 pp. <https://doi.org/10.4095/295753>.

Crow, H.L., Alpay, S., Hinton, M.J., Knight, R.D., Oldenborger, G.A., Percival, J.B., Pugin, A.J.M. and Pelchat, P., 2017. Geophysical, geotechnical, geochemical, and mineralogical data sets collected in Champlain Sea sediments in the Municipality of Pontiac, Québec. Geological Survey of Canada, Open File, Natural Resources Canada, 1-51 pp. <https://doi.org/10.4095/301664>.
